# Genetic analyses of tropical maize lines under artificial infestation of fall armyworm and foliar diseases under optimum conditions

**DOI:** 10.3389/fpls.2023.1086757

**Published:** 2023-01-20

**Authors:** Isaac Kamweru, Yoseph Beyene, Anani Y. Bruce, Dan Makumbi, Victor O. Adetimirin, Paulino Pérez-Rodríguez, Fernando Toledo, Jose Crossa, Boddupalli M. Prasanna, Manje Gowda

**Affiliations:** ^1^ International Maize and Wheat Improvement Center (CIMMYT), Nairobi, Kenya; ^2^ Pan African University, Life and Earth Sciences Institute (Including Health and Agriculture), Ibadan, Nigeria; ^3^ Department of Crop and Horticultural Sciences, University of Ibadan, Ibadan, Nigeria; ^4^ Colegio de Postgraduados, Montecillo, Mexico; ^5^ Biometrics and Statistics Unit, International Maize and Wheat Improvement Center (CIMMYT), Texcoco, Mexico

**Keywords:** maize, fall army worm, host plant resistance, general combining ability (GCA), specific combining ability (SCA), hybrid prediction

## Abstract

Development and deployment of high-yielding maize varieties with native resistance to Fall armyworm (FAW), turcicum leaf blight (TLB), and gray leaf spot (GLS) infestation is critical for addressing the food insecurity in sub-Saharan Africa. The objectives of this study were to determine the inheritance of resistance for FAW, identity hybrids which in addition to FAW resistance, also show resistance to TLB and GLS, and investigate the usefulness of models based on general combining ability (GCA) and SNP markers in predicting the performance of new untested hybrids. Half-diallel mating scheme was used to generate 105 F_1_ hybrids from 15 parents and another 55 F_1_ hybrids from 11 parents. These were evaluated in two experiments, each with commercial checks in multiple locations under FAW artificial infestation and optimum management in Kenya. Under artificial FAW infestation, significant mean squares among hybrids and hybrids x environment were observed for most traits in both experiments, including at least one of the three assessments carried out for foliar damage caused by FAW. Interaction of GCA x environment and specific combining ability (SCA) x environment interactions were significant for all traits under FAW infestation and optimal conditions. Moderate to high heritability estimates were observed for GY under both management conditions. Correlation between GY and two of the three scorings (one and three weeks after infestation) for foliar damage caused by FAW were negative (-0.27 and -0.38) and significant. Positive and significant correlation (0.84) was observed between FAW-inflicted ear damage and the percentage of rotten ears. We identified many superior-performing hybrids compared to the best commercial checks for both GY and FAW resistance associated traits. Inbred lines CML312, CML567, CML488, DTPYC9-F46-1-2-1-2, CKDHL164288, CKDHL166062, and CLRCY039 had significant and positive GCA for GY (positive) and FAW resistance-associated traits (negative). CML567 was a parent in four of the top ten hybrids under optimum and FAW conditions. Both additive and non-additive gene action were important in the inheritance of FAW resistance. Both GCA and marker-based models showed high correlation with field performance, but marker-based models exhibited considerably higher correlation. The best performing hybrids identified in this study could be used as potential single cross testers in the development of three-way FAW resistance hybrids. Overall, our results provide insights that help breeders to design effective breeding strategies to develop FAW resistant hybrids that are high yielding under FAW and optimum conditions.

## Introduction

Maize (*Zea mays* L.) is an important cereal crop to the global food, feed, fuel, and fiber system. Its transition from a peasant subsistence food crop to a major player in the local and national food economies of sub-Saharan Africa (SSA) illustrates its importance to the food security of the sub-region (Smale et al., 2013). Maize production in SSA however lags behind an ever-increasing consumer demand (Ittersum et al., 2016) due to frequent biotic and abiotic stresses that often occur together (Badu-Apraku and Fakorede, 2017). The recent outbreak of Fall armyworm [FAW; *Spodoptera frugiperda* (J.E. Smith)] insect pest in almost all countries of SSA (Feldmann et al., 2019) is projected to worsen the current food and income insecurity status of millions of smallholder farmers who derive their livelihoods from maize cultivation (Abrahams et al., 2017). FAW infests more than 353 host plant species across 76 families, the pest preferentially feeds on maize, rice, wheat, sorghum, and sugarcane, among other economic crop plants of the grass family (Montezano et al., 2018). In favorable climatic conditions, FAW can establish itself as a multi-generational pest of economic importance (Zacarias, 2020) due to its voracious appetite for a wide host range, high fecundity, and long-distance migratory capabilities that extend its geographical range (Feldmann et al., 2019). The trend of invasion of FAW across continents such as Africa (FAO, 2019), Asia (Lee et al., 2020) and Australia (Maino et al., 2021) puts other regions where FAW is currently absent such as Europe at high risk of an invasion (Paudel Timilsena et al., 2022) that may precipitate a global food crisis.

In SSA, maize yield losses ranging from 12 – 58% have been attributed to FAW infestation (Baudron et al., 2019; Kumela et al., 2019; Chimweta et al., 2020; De Groote et al., 2020), amounting to economic losses of US$159 - US$177 million annually (Rwomushana et al., 2018). Majority of the people in SSA who subsist on maize-based diets are also at risk of developing health disorders as presence of larval frass on FAW-damaged kernels create conditions that favor the growth of mold pathogens such as *Fusarium verticillioides* and *Aspergillus flavus* which produce mycotoxins poisonous to humans (Williams et al., 2018). Overall reduction of maize grain quality and quantity due to FAW destruction adversely impact the contribution of the maize crop to the agricultural sector which is the mainstay of most economies in SSA (Diao et al., 2007).

Considering the high potential losses caused by FAW infestation on major food crops, there is a strong need to implement various control strategies at regional, national and farmer level. Application of synthetic pesticides (Matova et al., 2020) and cultivation of transgenic *Bt.* (*Bacillus thuringiensis)* maize with activity against FAW (Botha et al., 2019) provide an immediate effective solution to FAW destruction. Different concerns have, however, emerged regarding the safety profile, availability, efficacy, cost-effectiveness and practicality for use of these options (Njuguna et al., 2021). Few transgenic maize varieties, for example, have been approved for use in most countries in SSA due to lack of biotechnology regulatory framework, political hurdles and consumer preference challenges (ISAAA, 2018). Indiscriminate application of synthetic pesticides in the control of FAW also raise toxicity concerns to humans (Tambo et al., 2020), environment (Togola et al., 2018), and non-target organisms such as insect-pollinators, beneficial arthropods, and aquatic life (Farrar et al., 2018). There have also been reports on the development of resistance by FAW to pesticides and transgenic maize (Storer et al., 2012; Farias et al., 2014; Schlum et al., 2021). Additionally, the effectiveness of eco-friendly cultural (Harrison et al., 2019) and biorational (Bateman et al., 2018) FAW control approaches are reduced at high pest populations.

The prevailing point of view is that the deployment of an integrated FAW management framework that includes utilization of maize germplasm with native resistance to FAW leaf feeding damage could be an effective long-term control strategy that promotes the sustainability of the broader agricultural ecosystem (Prasanna et al., 2022). Host plant resistance breeding to FAW in maize provides a cost-effective, locally accessible, seed-based control option that is scalable and compatible with chemical and biological control methods (Hruska, 2019). Loss of photosynthetic leaf area in maize susceptible to FAW and fungal pathogens such as *Exserohilum turcicum* that causes the turcicum leaf blight (TLB) disease, and *Cercospora zeae-maydis* that causes the gray leaf spot (GLS) disease adversely impact grain yield (Tefferi et al., 1996). According to Jakhar et al. (2017) and Nzuve et al. (2014), deploying host plant resistance to mitigate yield and economic losses associated with TLB and GLS foliar diseases provides an eco-friendly and cost-effective alternative to fungicide control.

Developing maize hybrids that are high yielding under FAW infestation, in addition to TLB and GLS, which are other prevalent diseases in many countries could be the most effective approach for achieving maize-based food self-sufficiency in SSA. Combining all the favorable alleles associated with increased yield potential and resistance to FAW, GLS, and TLB infestation in one or a few hybrids is, however, challenging due to paucity of resistant germplasm. Furthermore, many underlying genes whose effects are small but cumulative are known to regulate complex traits in maize such as grain yield (Wallace et al., 2014), resistance to FAW (Badji et al., 2020; Kamweru et al., 2022), GLS (Kibe et al., 2020) and TLB (Jakhar et al., 2017) foliar diseases. An in-depth understanding on the genetic differences for responses to FAW resistance associated traits like foliar damage (FD) and ear damage (ED), GY, and other agronomic traits, as well as the type of gene action regulating the inheritance of resistance to FAW, GLS and TLB is useful in prioritizing inbred lines to be used as parents in hybrid development.

Evaluation of all possible cross combinations generated in diallel mating schemes utilized in hybrid maize development has practical limitations. More efficient methods are, therefore, required to select for desirable traits among the parental lines and predict hybrid performance in a time and cost-effective manner (Heslot et al., 2013). For traits predominantly controlled by additive gene effects, GCA-based predictions are promising and could be utilized to choose few combinations with high performance, which improve the efficiency of hybrid maize breeding (Nyaga et al., 2020). Higher prediction efficiency has been reported with the incorporation of molecular information in predicting the performance of hybrids (Wang et al., 2020). The objectives of the present study were to investigate the genetic effects controlling GY and other important secondary traits under optimum and artificial FAW infestation conditions, identify superior single-cross hybrids under FAW-infested and non-infested conditions, and assess the efficiency of general combining ability (GCA) and molecular marker-based prediction of hybrid performance.

## Materials and methods

### Plant material and experimental design

A total of 26 inbred parents were used for this study. The lines were selected from the screening of over 2,000 inbred lines developed by CIMMYT using the double haploid (DH) technology. Inbred lines maintained by CIMMYT and obtained from different source germplasm were selected for various attributes including resistance to diseases such as maize lethal necrosis disease (MLN), FAW, GLS, TLB and drought tolerance (**Table 1**).

**Table 1:** List of the 28 parental inbred lines used to generate 185 F_1_ hybrids evaluated in two diallel experiments.

No.	Code	Seed color	Attribute
Experiment I			
1	CKDHL120566	White	FAW tolerant
2	CKDHL121288	White	FAW tolerant
3	CKDHL164260	White	FAW tolerant
4	CKDHL164271	White	FAW tolerant
5	CKDHL164288	White	FAW tolerant
6	CKDHL166062	White	FAW tolerant
7	CKDHL166068	White	FAW tolerant
8	CKDHL166075	White	FAW tolerant
9	CKLMARSI0183	White	Drought tolerant
10	CKLTI0344	White	Temperate introgressed, good yield
11	CML488	White	FAW tolerant, GLS resistant
12	CML567	White	Drought tolerant, GLS and TLB resistant
13	DTPYC9-F46-1-2-1-2	Yellow	MLN tolerant, SCMV tolerant
14	KS23-6	Yellow	MLN resistant
15	WMA2001	White	Drought tolerant
16	CKDHL164290	White	FAW tolerant
17	CML312	White	Good GCA for drought tolerant
Experiment II			
1	CKDHL0089	White	FAW tolerant, MLN tolerant
2	CKDHL120348	White	FAW tolerant
3	CKDHL120668	White	FAW tolerant
4	CKDHL121320	White	MLN tolerant
5	CKDHL166087	White	FAW tolerant
6	CKDHL166091	White	FAW tolerant
7	CKDHL166092	White	FAW tolerant
8	CKLMLN140377	White	MLN tolerant
9	CKLMLN140538	White	MLN tolerant
10	CLRCY039	Yellow	Drought tolerant, MLN tolerant
11	CML494	White	MLN tolerant, Low N stress tolerant

MLN, maize lethal necrosis; GLS, Gray leaf spot; TLB, Turcicum leaf blight.

A half-diallel mating scheme was used to generate 136 hybrids from 17 parental lines, however, we fail to get the seeds for few combinations, therefore, for the final analyses we included only the parental lines which has all combinations. For the final analyses 105 F_1_ hybrids from 15 parental lines in experiment I and 55 F_1_ hybrids from 11 parental lines in experiment II. All hybrids in the two sets of diallel were evaluated alongside nine commercial checks (DH04, DK777, DK8031, Duma43, H516, PAN3MO1, Pioneer30G19, WE1101, and WH505) under artificial FAW infestation in Kiboko for three seasons and optimum conditions in Kakamega, Kiboko, Embu, and Kirinyaga in Kenya (**Table 2**). Most of the selected commercial checks are susceptible to FAW infestation. These two sets of diallel experiments were evaluated for three seasons under artificial FAW infestation conditions. The layout for both experiments followed an α-lattice design with two replications at each site. Entries were planted in two row plots, each 4 m long with 75 cm between rows. Two seeds were planted per hill at a spacing of 25 cm. Stands were thinned to one plant per hill three weeks after emergence to obtain a final plant population density of 53,333 plants per hectare. All the recommended agronomic management practices were implemented up till physiological maturity when the ears were harvested.

**Table 2:** Description of locations used for the evaluation.

Location	Longitude	Latitude	Max. (°C)	Min. (°C)	Rainfall (mm)	Altitude (masl)	Soil type
Kiboko	37.75`E	2.15`S	28.6	16.5	530	950	Sandy clay
Kakamega	34.45`SE	0.16`N	28.6	12.8	1915	1585	Sandy loam
Kirinyaga	37.19`E	0.34`S	24.0	18.0	1500	1282	Clay loam
Embu	37.41`E	0.45`S	25.0	14.1	1200	1510	Clay loam

Masl, meters above sea level; mm, millimeter.

### Artificial FAW infestation

FAW colonies were reared using artificial diet as described by Prasanna et al. (2018) and under ambient laboratory conditions (temperature of 27 ± 1^0^C, 12:12 h light/dark, and relative humidity of 70 ± 5%) at the Kenya Agricultural and Livestock Research Organization (KALRO) insectary located at Katumani (1^0^35`S, 37^0^15`E, 1,610 masl). Trials under artificial FAW infestation were set up in insect-proof screen-houses at Kiboko. A paint brush was used to manually apply eight first instar FAW larvae at the furl and whorl leaves of each plant at 3-leaf (V3) phenological stage of maize growth and development (Nielsen, 2014). Infestation at V3 maize growth stage favours the conditioning of FAW larvae to host environment in terms of feeding on the soft leaf tissues and survival. All plots were infested on the same day to ensure uniformity of infestation.

### Phenotypic evaluations

In trials under FAW artificial infestation, ten plants per plot were visually rated for foliar damage at 7 (FD1), 14 (FD2) and 21 (FD3) days after artificial FAW infestation. A visual rating scale of 1 to 9, described by Prasanna et al. (2018), was used to score leaf feeding damage. On this scale, 1 = no visible damage, 2 = few short holes on several leaves, 3 = short holes on several leaves, 4 = several leaves with short holes and a few long lesions, 5 = several holes with long lesions, 6 = several leaves with lesions< 2.5 cm, 7 = long lesions common on one half of the leaves, 8 = long lesions common on one half to two thirds of leaves, and 9 = severe damage, most leaves with long lesions and complete defoliation. For each plot, mean FD1, FD2 and FD3 was computed. Other data collected were days to anthesis (AD; days from planting to 50% pollen shed), days to silking (SD; days from planting to 50% silking), anthesis to silking interval (ASI; difference between SD and AD), plant height (PH; distance in cm from the ground to the top of the tassel), ear height (EH; distance in cm from the base of the plant to the main ear-bearing node), and ears per plant (EPP; ratio of number of ears to number of plants harvested per plot). Ear position (EPO), was calculated as the ratio of EH to PH. Ear damage (ED; damage caused by the FAW larvae on maize kernels once it gains entry into the developing ears) was rated on a scale of 1-9, where, 1 = no visible damage to the ear, 2 = damage to a few kernels (<5) or less than 5% damage to an ear, 3 = damage to a few kernels (6-15) or less than 10% damage to an ear, 4 = damage to (16-30) kernels or less than 15% damage to an ear, 5 = damage to (31-50) kernels or less than 25% damage to an ear, 6 = damage to (51-75) kernels or more than 35% but less than 50% damage to an ear, 7 = damage to (76-100) kernels or more than 50% but less than 60% damage to an ear, 8 = damage to >100 kernels or more than 60% but less than 100% damage to an ear, 9 = almost 100% damage to an ear. FAW-damaged maize kernels are predisposed to mold pathogens that cause rots to develop on individual kernels or part of the ear (Prasanna et al., 2018; Kamweru et al., 2022). Ear rot (ER; determined by assessing the percent area of each ear covered by rot symptoms) was assessed using a rating scale of 1-9, where 1 = 0%; 2 = 1-20%; 3 = 21-30%; 4 = 31-40%; 5 = 41-50%; 6 = 51-60%; 7 = 61-70%; 8 = 71-80%; and 9 = 81-100%. Moisture content (MOI) in percentage of the shelled grains at harvest was determined using a hand-held moisture meter. Grain yield (GY) was obtained from the shelled grain weight per plot, converted to tons per hectare (t/ha) and adjusted to 12.5% moisture content. FAW larvae chew an exit hole on the maize stem before pupation and moth emergence. The leaves on the maize stalks were removed and the number of FAW exit holes (EXHL) were counted for ten representative plants in a plot. Average EXHL was computed on plot basis. Tunneling length (TLGTH) along the split maize stalk was measured in cm for ten plants per plot and the average TLGTH was expressed in percentage. Trials under optimum management conditions were left to natural GLS and TLB infection in disease hot spot locations (Kakamega, Embu, and Kirinyaga). Long cigar-shaped, gray to tan colored lesions on the leaves were used to identify TLB infected genotypes while small gray to brown necrotic spots with halos were the characteristic symptoms used to identify GLS infected plants (CIMMYT, 2004). Reaction to TLB and GLS diseases were visually rated per plant using a scale of 1-9 as described by Munkvold et al. (2001). A score of 1 denoted 0% of the leaf area covered by disease symptoms 2 = less than1%, 3 = 1-3%, 4 = 4-6%, 5 = 7-12%, 6 = 13-25%, 7 = 26-50%, 8 = 51-75%, and 9 denoting approximate disease symptom coverage of 76-100%. Scoring started at mid-silking stage when differences among plots for reaction to these diseases were noticeable and was repeated at hard dough developmental stage (Derera et al., 2008). In addition to GLS and TLB data, agronomic data were collected on AD, SD, ASI, PH, EH, EPO, EPP, ER, MOI, and GY traits.

### Data analysis

For the analyses, each management at each of the locations/seasons was treated as an environment. Since FAW resistance traits and foliar disease traits were scored on an ordinal scale, data were tested for fulfilment of the basic assumptions for valid statistical analysis. In the process of quality check, detected outliers were excluded from further analyses. META-R (Multi Environment Trait Analysis R) software was used to get best linear unbiased predictions (BLUPs) and best linear unbiased estimates (BLUEs) for each hybrid (Alvarado et al., 2015).

The checks were excluded from the diallel analysis in both the experiments. The GCA effects of the parental lines and the SCA effects of F_1_ hybrids, as well as their mean squares under each and across environment but within each management were estimated according to Griffing’s Method 4 model 1 (fixed effects; Griffing, 1956). Data were analyzed using the AGDR-R version 4 software (Rodríguez et al., 2015). Analyses were performed based on the following model:


$$Y_{ii'k}=\;µ\;+\;E_{k}+G_{i}+\;G_{i'}+\;S_{ii'}+\;{\left(EG\right)}_{ik}+{\left(EG\right)}_{i'k}+{\left(ES\right)}_{ii'k}+e_{ii'k}$$

where Y*_ii_*_´_ *_k_* is the performance of the single cross hybrid (*i*_x_ *i*´) in the *k*th environment; µ is the overall mean; E*_k_* is the *k*th environment effect; G*_i_*, G*_i_*_´_, and S*_ii_*_´_ are GCA and SCA effects (Griffing, 1956); (EG)*_ik_* and (EG)*_i_*_´_ *_k_* are GCA effects of i and i´ parents and their interaction with environment, respectively; (ES)*_i_*_i´_ *_k_* is SCA interaction with environment; and e*_i_*_i´_ *_k_* is the error term. In the combined analysis, the mean squares for hybrid and environment were tested against the mean squares for genotype-by-environment (GE) interaction as error terms, while GE mean squares were tested against pooled error. Similarly, the significance of GCA and SCA sources of variation was determined using the corresponding interactions with the E as error terms. Error mean squares used to test the significance of GCA and SCA interactions with E were obtained by dividing the pooled error mean squares from the ANOVA by the number of replications, because the combining ability mean squares were calculated based on entry means (Griffing, 1956; Dabholkar, 1999; Ferreira et al., 2004). The GCA and SCA effects were tested for significance by t-test, using standard errors of GCA and SCA effects, respectively. GCA ( $\sigma_{\text{GCA}}^{2}$ ) and SCA ( $\sigma_{\text{SCA}}^{2}$ )variance components were estimated from the corresponding combining ability effects. The relative importance of GCA and SCA effects for each trait was determined using Bakers ( ${\mathrm{2\sigma}}_{\text{GCA}}^{2}$ /( ${\mathrm{2\sigma}}_{\text{GCA}}^{2}+\sigma_{\text{SCA}}^{2}$ )) ratio (Baker, 1978). Heritability was estimated on an entry-mean basis from the variance components as the ratio of the genotypic to phenotypic variance. Pearson’s correlation coefficients were calculated between pairs of agronomic traits evaluated under FAW and optimum management conditions using BLUPs across environments. In addition we investigated the correlation between F_1_ hybrids and the sum of GCA effects of both the parents r(GCA, F_1_P) using a leave-one-hybrid-out cross validation procedure as described by Schrag et al. (2009). All analyses were performed using ASReml-R software version 3.0 (Butler et al., 2009).

Among the 15 inbred lines from experiment I, DNA of 13 lines was extracted from 3-4 weeks old seedlings and Diversity Array Technology (DArT) marker platform was used to develop 42,376 single nucleotide polymorphisms (SNPs). Trait Analysis by Association, Evolution and Linkage (TaSSEL) software (Bradbury et al., 2007) was used to summarize genotype data by site, determine the allele frequencies and implement quality screening. The distribution of the proportion of missing values were plotted in **Supplementary Figure S1**. After removing markers with more than 15% of missing values, 18,074 markers were retained for downstream analysis. We imputed missing values using observed allelic frequencies and removed monomorphic markers: a total of 11,121 SNPs were retained. A stringent quality filtering criteria was implemented where SNP variants that were monomorphic and which, had minor allele frequency of<0.05 were removed. The distribution of markers with MAF (>0.05) are shown in **Supplementary Figure S2**. A total of 10,473 high quality SNPs were retained for marker-based prediction of hybrid performance. The linear model used for hybrid performance predictions is according to Technow et al. (2014) as follows:

*y*=*Z*_1_ *g*_1_+*Z*_2_ *g*_2_+*Z*_*h*_ *h*+*ϵ* . where *y* . the response vector (i.e., the adjusted hybrids’ phenotypic information), *g*_1_ the vector of random effects due to the GCA of parental lines 1, *g*_2_ the vector of random effects due to the GCA of markers for parental lines 2 and *h* the vector that includes SCA random effects and denotes the interaction effects between parental lines 1 and 2 for the hybrids. *Z*_1_, *Z*_2_, *Z*_*H*_ e incidence matrices that relate *y* $g_{1},\;g_{2},\;h,\text{ with }g_{1}\sim N\left(0,{\text{σ}}_{1}^{2}G_{1}\right)$ here ${\text{σ}}_{1}^{2}$ ${\text{σ}}_{H}^{2}$ e variance components associated with GCA for parents 1, parent 2, and SCA; and *G*_1_, *G*_2_ d *H* e relationship matrices for parental lines 1 and 2, and hybrids, respectively. Finally, $\epsilon \sim N\left(0,\sigma_{\text{ϵ}}^{2}I\right)$ here $\sigma_{\text{ϵ}}^{2}$ the variance associated with the residuals.

The relationship matrices *G*_1_ d *G*_2_ re computed using the markers (Lopez-Cruz et al., 2015). The elements of matrix *H* n be obtained directly from matrices *G*_1_ d *G*_2_  (Bernardo, 2002; Technow et al., 2014).


$$\begin{array}{c} Cov\left(h_{ij},h_{i\prime j\prime }\right)\\ =Cov\left(g_{1}i\;,g_{2}i^{\prime }\right)\times Cov\left(g_{1}j\;,\;g_{2}j^{\prime }\right)\propto G_{1}ii^{\prime }\times G_{2}jj^{\prime } \end{array}$$

Where *G*_1*ii* ^′^_ *G*_2*jj* ^′^_ e entries from *G*_1_ d *G*_2_, spectively. In compact notation, matrix *H* r all possible crosses is obtained as the Kronecker product of *G*_1_ d *G*_2_ ,hat is, *H*=*G*_1_⊗*G*_2_ (Covarrubias-Pazaran, 2016). The marker-based prediction model was fitted using the Bayesian Generalized Linear Regression (BGLR) package in R environment (Pérez & De Los Campos, 2014). For marker-based prediction, we mimicked a common problem faced by breeders when testing new hybrids using limited field trials by predicting the performance of newly developed or missing combination of hybrids. Five-fold cross validations were applied for all traits under FAW infestation and optimum management conditions.

## Results

### ANOVA under artificial FAW infestation

Analyses of variance revealed significant mean squares among hybrids for most traits measured across environments in both the experiments except for foliar damage scores at 14 (FD2) and 21(FD3) days after artificial FAW infestation in experiment I and number of exit holes (EXHL) in experiment II (**Table 5**). Mean squares due to environment were significant (P<0.01) for all traits in experiments I and II. Hybrid x environment interactions (GEI) were significant (*P*<0.05 – *P*<0.01) for all traits in both experiments, except days to anthesis, foliar damage scores at 7 (FD1) and 14 (FD2) days after FAW infestation and number of exit holes in experiment I. In both experiments, mean squares for hybrids were higher than mean squares obtained for hybrid x environment interaction (HxE) for all traits, except EXHL and tunnel length (TLGTH) in experiment II. Following the partitioning of hybrid mean squares into GCA and SCA mean squares, variances due to GCA mean squares were found to be significant for all ten traits evaluated under FAW infestation in both the experiments. Mean squares due to SCA were significant for most of the traits except EXHL and TLGTH in experiment I and FD1 and FD2 in experiment II. All traits except GY in experiment I and EXHL in experiment II had significant GCA x environment interaction mean squares. SCA x environment interaction mean squares were significant for GY, PH, and ER traits in experiment I and all traits in experiment II. The proportion of GCA sum of squares was larger than SCA sum of squares for all traits evaluated under FAW infestation in both the experiments, except for GY in experiment II for which SCA mean square was higher (**Table 3**). The ratio of GCA effects to the total genetic effects for all traits evaluated under artificial FAW infestation conditions ranged from 0.83 (GY) to 0.94 (AD) in experiment I and from 0.55 (GY) to 0.94 (ED) in experiment II.

**Table 3:** Mean squares for grain yield and other FAW resistance associated traits of 160 medium maturing tropical maize hybrids evaluated in two diallel experiments under artificial FAW infestation in Kiboko, Kenya.

Source	DF^‡^	GY	AD	PH	FD1	FD2	FD3	ED	EXHL	ER	TLGTH
Experiment I											
Environments, E	2/1	817.11**	292.35**	192098.19**	379.89**	342.09**	286.41**	34.83**	1466.24**	590.97**	18354.39**
Rep (Env), R	3/2	31.66**	10.64**	3270.01**	9.35**	9.49**	9.96**	3.90**	16.66**	2494.08**	339.88**
Hybrids, H	104	8.77**	24.88**	1843.15**	0.61*	0.54ns	0.67ns	2.55**	2.78*	405.22**	54.39**
GCA	14	18.47**	98.35**	6556.10**	1.35**	1.36**	2.37**	7.41**	4.85**	1427.09**	165.0**
SCA	90	7.64**	13.45**	1110.02**	0.50*	0.41*	0.41*	1.79*	2.42ns	256.66**	36.89ns
HxE	208/104	4.28**	6.79ns	562.03**	0.62ns	0.49ns	0.65*	1.61*	2.50ns	200.0**	42.04*
GCA x E	28/14	9.02**	11.23**	1158.16**	1.22**	1.09**	1.14**	3.87**	2.63ns	310.22**	74.03**
SCA x E	180/90	3.49**	6.10ns	469.30**	0.53ns	0.39ns	0.57ns	1.30ns	2.53ns	182.87**	37.44ns
Error	150/99	2.96	6.41	445.00	0.51	0.45	0.53	1.24	2.75	136.16	29.96
Heritability		0.53	0.63	0.80	0.32	0.33	0.35	0.65	0.31	0.54	0.41
2GCA/(2GCA+SCA)		0.83	0.94	0.92	0.84	0.87	0.92	0.89	0.80	0.92	0.90
Experiment II											
Environments, E	2/1	455.22**	654.10**	122749.41**	310.43**	126.60**	158.12**	102.49**	526.77**	1607.16**	12319.42**
Rep (Env), R	3/2	14.34**	32.76**	10216.06**	6.05**	3.09**	3.24**	1.02*	8.34**	10.62ns	38.16**
Hybrids, H	54	5.61**	15.55**	1962.32**	0.66*	0.65*	0.84**	4.12**	1.49ns	403.87**	53.51**
GCA	10	3.77**	38.14**	3759.08**	1.40**	1.25**	1.73**	15.95**	1.81*	1278.20**	114.18**
SCA	44	6.06**	10.43**	1553.96**	0.50ns	0.51ns	0.64*	2.05**	1.39*	265.54**	37.19**
HxE	108/54	3.33**	5.92**	799.13**	0.62**	0.56*	0.66*	2.29**	2.16**	177.35**	68.95**
GCA x E	20/10	1.91ns	10.11**	864.78**	0.89*	0.77**	1.18**	5.44**	5.52**	229.42**	164.18**
SCA x E	88/44	3.65**	4.96**	784.21**	0.56**	0.51*	0.55*	1.11**	1.43*	138.82**	50.20**
Error	106/66	2.51	3.51	416.98	0.38	0.31	0.35	0.58	0.88	75.68	18.49
Heritability		0.61	0.82	0.89	0.49	0.38	0.39	0.51	0.54	0.56	0.71
2GCA/(2GCA+SCA)		0.55	0.88	0.83	0.85	0.83	0.84	0.94	0.72	0.91	0.86

*, **, significant at the 0.05 and 0.01 probability level, respectively; ‡ DF, degrees of freedom; “n1/n2” indicates degrees of freedom for GY, AD, PH, FD1, FD2 and FD3 (n1, for three environments) and for ED, EXHL, ER and TLGTH (n2, for two environments), respectively. GCA, general combining ability effects; SCA, specific combining ability effects. GY, grain yield; AD, days to anthesis; PH, plant height; FD1, FD2, FD3, mean foliar damage scores at 7, 14 and 21 days after artificial infestation, respectively; ED, ear damage; EXHL, number of exit holes; TLGTH, tunneling length; ER, number of rotten ears in %.

### ANOVA under optimum conditions

Analyses of variance under optimum conditions revealed highly significant differences (P<0.05-*P*<0.01) among hybrids for all traits in both experiments (*P*<0.05; **Table 4**). Mean squares for GCA and SCA were highly significant (*P*<0.01) for all ten traits studied in two experiments. Differences among the environments were also highly significant (*P*<0.01) for all traits in both the experiments. All traits had significant GCA x environment and SCA x environment interaction mean squares, but the magnitudes were consistently smaller than the respective GCA and SCA mean squares in both experiments (**Table 5**).

**Table 4:** Mean performance of best 10 F_1_ experimental hybrids for grain yield and other agronomic traits under FAW infestation and optimum conditions in two diallel experiments.

Experiment I	FAW infestation					Optimum			
Hybrids	GY (t/ha)	AD (days)	FD2 (1-9)	FD3 (1-9)	ED (1-9)	GY (t/ha)	AD (days)	GLS (1-9)	TLB (1-9)
CKDHL164260/CML567	7.50	63.34	3.00	6.57	2.93	6.79	69.59	2.51	3.34
CML567/KS23-6	7.33	63.51	4.22	6.03	2.92	7.72	73.45	3.04	2.83
CML312/CKDHL164288	6.99	62.69	4.50	5.93	3.18	8.86	71.49	3.98	3.90
CML567/CKDHL166068	6.79	60.98	4.31	5.05	3.08	8.84	71.54	2.45	2.95
CML312/CML488	6.55	60.96	4.01	5.69	2.19	8.37	69.31	2.99	3.36
CKDHL164288/CML567	6.45	64.15	3.81	6.05	5.25	9.69	72.20	3.01	3.32
CML488/CML567	6.43	63.96	4.67	5.54	2.08	7.08	73.49	1.94	2.37
CML312/CKDHL166062	6.42	63.63	3.92	6.06	2.48	8.25	71.62	2.47	3.03
CML312/CKDHL164271	6.37	61.67	4.04	5.84	2.68	9.10	68.73	2.99	2.41
CML567/CKDHL164271	6.34	61.80	4.29	5.79	2.40	9.35	70.30	1.97	2.88
Mean: top ten hybrids	6.72	62.67	4.08	5.86	2.92	8.41	71.17	2.74	3.06
All hybrids	4.47	61.86	4.05	5.97	3.41	6.91	69.57	2.08	3.37
Checks	4.08	61.37	3.96	6.07	3.93	6.98	70.55	1.99	3.40
Experiment II									
CKDHL120348/CKDHL166087	8.18	66.17	5.57	5.48	3.99	7.68	75.49	3.03	3.68
CKDHL121320/CLRCY039	8.17	66.59	4.38	4.19	2.20	9.54	75.06	3.02	3.99
CKDHL166087/CLRCY039	7.69	65.22	4.57	4.45	1.96	8.31	74.66	3.10	3.36
CKDHL89/CKLMLN140377	7.63	65.07	5.00	4.97	3.05	9.20	73.81	3.09	4.67
CKDHL121320/CKLMLN140538	7.49	66.04	5.26	5.47	2.98	8.14	72.20	3.10	3.67
CKLMLN140377/CKLMLN140538	7.40	63.91	5.08	5.06	3.37	7.49	71.34	3.04	3.67
CKDHL89/CKDHL166091	7.37	66.79	5.12	5.29	2.54	5.16	75.98	3.08	3.66
CKDHL120668/CKDHL166087	7.24	65.89	4.74	5.01	2.56	7.57	73.49	3.08	3.66
CKDHL120348/CKDHL166091	7.07	65.57	5.03	5.39	3.62	7.41	74.64	3.67	3.65
CKDHL166091/CML494	6.99	66.77	5.34	5.45	2.71	6.51	74.12	2.82	5.32
Mean: top ten hybrids	7.52	65.80	5.01	5.08	2.90	7.70	74.08	3.10	3.93
All hybrids	5.91	66.21	4.99	5.37	3.35	7.12	74.10	1.67	4.06
Checks	3.74	63.94	5.27	5.37	4.57	6.77	71.69	3.18	3.34

GY, grain yield; AD, days to 50% anthesis; FD2, FD3, foliar damage scores 14 and 21 days after artificial infestation respectively; ED, ear damage; TLB, turcicum leaf blight; GLS, gray leaf spot.

**Table 5:** Mean squares for grain yield and other agronomic traits of 160 medium maturing tropical maize hybrids evaluated in two diallel experiments under optimal conditions in three to five locations in Kenya.

Source	DF^‡^	GY	AD	ASI	PH	EH	TLB	GLS	EPO	EPP	ER
Experiment I											
Environments, E	3/2	932.79**	14973.36**	128.10**	65822.91**	14070.48**	58.82**	3.84**	0.8114**	0.122**	2901.63**
Rep (Env), R	4/3	1.01*	21.54**	1.27*	21163.92**	559.11**	5.66**	0.05*	0.0008*	0.033**	35.33**
Hybrids, H	104	11.93**	42.47**	6.43**	1764.32**	613.62**	2.59**	1.64**	0.0065**	0.018*	101.63**
GCA	14	28.99**	228.96**	25.96**	7651.87**	2045.17**	11.01**	7.93**	0.0288**	0.040**	307.13**
SCA	90	9.27**	13.14**	3.43**	892.44**	390.94**	1.28*	0.67*	0.0030**	0.014*	69.66**
HxE	312/208	2.19**	3.72**	1.58*	196.93**	109.87**	1.35*	1.65**	0.0009*	0.017**	36.44**
GCA x E	42/28	6.19**	10.44**	2.53**	386.78**	276.80**	4.38**	8.12**	0.0024**	0.036**	69.29**
SCA x E	270/180	1.59**	4.15*	1.43*	181.61**	83.90*	0.88*	0.69*	0.0006*	0.014*	31.33**
Error	200/150	0.92	0.85	1.00	46.06	34.84	0.87	0.31	0.0003	0.010	26.20
Heritability		0.90	0.95	0.85	0.94	0.90	0.69	0.31	0.90	0.55	0.62
2GCA/(2GCA+SCA)		0.86	0.97	0.94	0.94	0.91	0.95	0.96	0.95	0.85	0.90
Experiment II											
Environments, E	4/2	555.25**	7880.08**	56.02**	119818.22**	89908.32**	6.45**	21.75**	0.4069**	0.157**	1306.17**
Rep (Env), R	5/3	22.68**	50.63**	0.93*	15739.11**	4468.36**	1.27**	0.55*	0.0076**	0.019*	177.97**
Hybrids, H	54	28.90**	30.96**	3.92**	2516.97**	1437.35**	0.74*	0.28*	0.0095**	0.046**	81.55**
GCA	10	76.55**	105.50**	13.99**	3774.29**	4673.85**	3.11**	0.76**	0.0462**	0.136**	148.35**
SCA	44	18.27**	14.02**	1.63**	2231.22**	701.78**	0.20*	0.17*	0.0012*	0.025*	66.37**
HxE	216/108	2.60**	4.78**	1.51**	419.68**	227.16**	0.38*	0.28*	0.0013*	0.016ns	49.38**
GCA x E	40/20	5.72**	6.42**	1.41**	512.14**	327.80**	0.72**	0.65**	0.0024*	0.020*	83.15**
SCA x E	176/88	1.90*	4.40**	1.53**	398.66**	204.29**	0.30*	0.19*	0.0011*	0.016*	41.70**
Error	180/108	1.01	3.01	1.12	104.30	68.07	0.18	0.13	0.0005	0.018	34.13
Heritability		0.91	0.88	0.70	0.91	0.92	0.49	0.71	0.91	0.61	0.42
2GCA/(2GCA+SCA)		0.89	0.94	0.94	0.77	0.93	0.97	0.90	0.99	0.91	0.82

*, and ** Significant at the 0.05 and 0.01 probability level, respectively. ‡ DF, degrees of freedom; “n1/n2” indicates degrees of freedom for GY, AD, PH, ASI, EH, EPO, EPP and ER (n1, for four environments in Exp I and five environments in Exp II) and for GLS and TLB (n2, for three environments in both experiments), respectively. GCA, general combining ability effects; SCA, specific combining ability effects. GY, grain yield; AD, days to 50% anthesis; ASI, anthesis to silking interval; PH, plant height; H, ear height; TLB, turcicum leaf blight; GLS, gray leaf spot; EPO, ear position; EPP, ears per plant; ER, ear rot in %.

### Hybrids performance and trait heritability

Under artificial FAW infestation and optimal conditions, frequency distributions of BLUPs for all traits evaluated in experiment I and II revealed normal to near-normal distributions except for GLS under optimum conditions (**Supplementary Figure S3**, **S4**). FD rating was continuously distributed from the resistance to the susceptible range. None of the hybrids expressed very high resistance (average score of 1) or very high susceptibility (average leaf damage score of 8-9) in both experiments. In experiment I, the FD ranged from 3.4 to 4.8 (Mean = 4.10) at 14 days after infestation and from 4.7 to 7.0 (Mean =5.90) at 21 days after infestation (**Table 3**). In experiment II, the range was 2.5 to 4.6 (Mean = 4.90) at 14 days after infestation and 4.6 to 6.4 (Mean = 5.40) at 21 days after infestation. In experiment I, the mean GY of the hybrids was 4.47 t/ha (range = 3.07 to 5.53 t/ha) under FAW infestation, while under optimum conditions, GY averaged 6.91 t/ha (range = 2.61 to 9.69 t/ha). In experiment II, the mean GY of the hybrids was 5.91 t/ha and 7.12 t/ha under FAW infestation and optimum conditions, respectively.

Under artificial FAW infestation conditions, heritability ranged from 0.31 (EXHL) to 0.80 (PH), and 0.38 (FD2) to 0.89 (PH) in experiment I and II, respectively. Moderately low (0.32-0.38) heritability estimates were observed for FD1, FD2, and FD3 while high heritability estimates were observed for traits such as PH (0.89), AD (0.82), and TLGTH (0.71). Moderately high heritability estimates were observed for GY under artificial FAW infestation conditions in experiment I (0.53) and II (0.61). The ratio of GCA effects to the total genetic effects (2GCA+SCA) ranged from 0.80 (EXHL) to 0.94 (AD) in experiment I and 0.55 (GY) to 0.94 (ED) experiment II.

Under optimum conditions, heritability ranged from 0.31 (GLS) to 0.95 (AD) in experiment I and 0.42 (ER) to 0.92 (EH) in experiment II. In experiment I, higher heritability (0.69) was observed for TLB compared to GLS (0.31) whereas in experiment II, TLB had a lower (0.49) heritability estimate in comparison to that of GLS (0.71). Heritability for GY under optimum conditions was high in both experiment I (0.90) and II (0.91). The ratio of GCA effects to the total genetic effects (2GCA+SCA) ranged from 0.85 (EPP) to 0.97 (AD) in experiment I and 0.77(PH) to 0.99 (EPO) in experiment II.

Among the experimental hybrids, two hybrids CKDHL164288/CKDHL164290 and CKDHL166068/CKDHL166075 were the poor performer with GY of<0.9 ton/ha and FD3 and COB ratings of >6.0 under artificial infestation of FAW. Whereas under optimum management, three hybrids CKDHL166068/CKDHL120566, CKDHL120566/CKDHL164290 and CKDHL164288/CKDHL120566 showed score of >6 for GLS and TLB which also serve as a susceptible check. Hybrid performance was ranked based on GY in trials evaluated under artificial FAW infestation and optimal conditions (**Table 4**). Under artificial FAW infestation conditions, the mean GY for the top ten hybrids was 6.7 t/ha in experiment I and 7.5 t/ha in experiment II. Under optimal conditions, the mean GY for the top ten hybrids was 8.40 and 7.70 t/ha in experiment I and II, respectively. The average GY for all commercial checks was poorer than the average GY for the top ten hybrids under artificial FAW infestation in both experiment I and II (4.08 vs 6.72 t/ha in experiment I; 3.74 vs 7.52 t/ha in experiment II). Among the commercial checks evaluated DUMA43 was the best with 5.04 t/ha under FAW infestation whereas WH505 was the best check under optimum conditions with 8.60 t/ha. In experiment I, CKDHL164260 x CML567 was the best hybrid under FAW infested conditions and produced 48% more GY than DUMA43 which was the best commercial check. In experiment II, the highest yielding hybrid under FAW infested conditions (CKDHL120348 x CKDHL166087) outyielded the best commercial check (DUMA43) by 62%. Under optimal conditions in both experiments I and II, seven hybrids outperformed WH505 which was the best commercial check. Foliar damage for the top 10 hybrids at 21 days after FAW infestation was 3% lower than the average for all commercial checks in experiment I, and 5% lower for the top 10 hybrids in experiment II (**Table 3**). Under artificial FAW infested conditions, the top ten yielding hybrids in both experiment I and II had, on average, reduced ED (2.9 vs. 4.6), fewer number of exit holes (0.6 vs. 1.3), shorter tunneling length (3.7 vs. 8.5) and a lower percentage of rotten ears (23 vs. 42) in comparison to the average for all commercial checks.

Under optimum conditions, differential expression of resistance to TLB and GLS diseases was observed among the hybrids. Although none of the hybrids exhibited a highly resistant or highly susceptible reaction to TLB and GLS diseases under natural infection, considerable skewness was observed towards the resistance range for GLS (**Supplementary Figure S3**, **S4**). The mean disease severity score was 3.4 and 4.0 for TLB and 2.1 and 1.7 for GLS, in experiment I and II, respectively. On average, the top ten hybrids in experiment I had lower TLB disease severity scores (Mean = 3.1) when compared to the mean for all checks (Mean = 3.4) while in experiment II, the average TLB disease severity score for the top ten hybrids was higher (3.9). The mean GLS score for the top ten hybrids in experiment I was higher (2.7) than that of checks (1.9) while in experiment II, mean GLS score for the top ten hybrids (3.1) was the same as the mean of all checks (3.1). Under FAW infested conditions, CML567 was one of the parents in six of the top 10 hybrid in experiment I, followed by CML312 (3), CKDHL164288 (2), and CKDHL164271 (2) whereas CKDHL166087 was involved in three of the top 10 hybrids in experiment II.

### Trait correlations under artificial FAW infestation and optimum conditions

**Figure 1:**
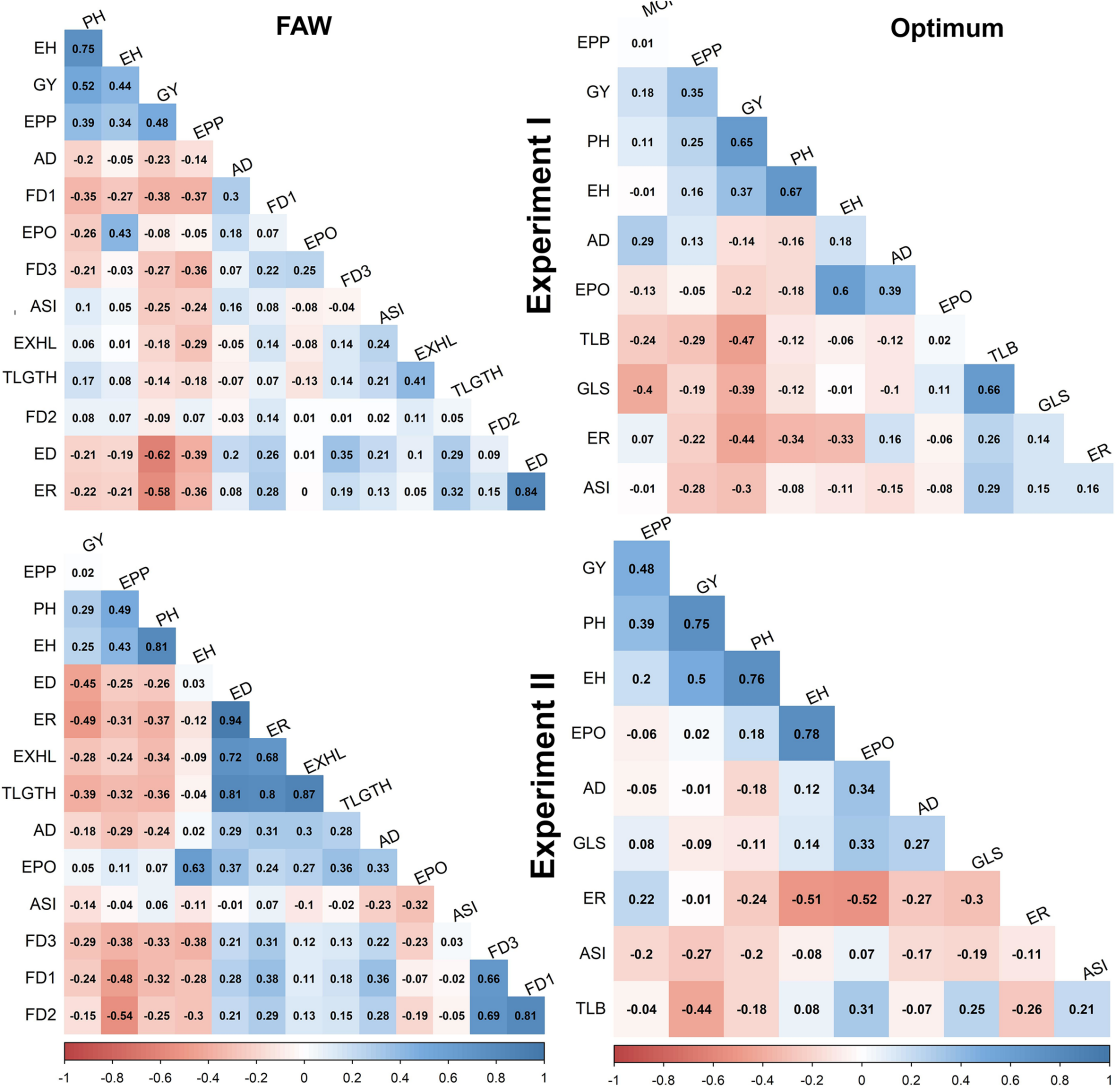
Trait correlations under FAW infestation and optimum management conditions in experiment I and II. The correlation level is color-coded according to the color key indicated on the scale. Correlations with >0.15 and >0.19 were significant at 0.05 and 0.01 levels, respectively. AD, days to 50% anthesis; ASI, anthesis to silking interval; ED, ear damage; PH, plant height; EH, ear height; GY, grain yield; FD1, FD2, FD3, mean leaf damage scores 7,14 and 21 days after artificial infestation respectively; t/ha, tons per hectare; TLB, turcicum leaf blight; GLS, grey leaf spot.; ER, ear rot; EXHL, number of exit holes; TLGTH, tunnel length; EPO, ear position; EPP, ears per plant; MOI, moisture content.

Under FAW infestation, GY was significantly correlated with all traits except TLGTH, FD2 and EPO in experiment I and EPP, EPO and ASI in experiment II (**Figure 1**). Significant correlations of GY were positive for PH, and EH (r = 0.25 to 0.52) and negative for all other traits in both the experiments. The highest negative correlation with GY were obtained with ED (r = -0.62 and ER (r = -0.58) in both the experiments. The correlation between ED and ER was significant and positive in both the experiments (r = 0.84 and 0.94). Significant and positive correlation (r = 0.41&0.87) was also observed between number of exit holes (EXHL) and cumulative tunneling length (TLGTH) as well as between FD1 and FD3 on one hand and ear damage and the percentage of rotten ears on the other. Foliar feeding damage at 14 days after FAW infestation was significantly correlated with ER but not with ED. The correlation between traits were consistent in both the experiments, except FD2 which is significantly negative correlation with GY in experiment II. Compared to experiment I, in experiment II strong positive significant correlations were observed between FD traits and ED as well as between EXHL, ER and TLGTH (**Figure 1**).

Under optimum conditions, positive and highly significant correlation was observed between GY and both EH (r = 0.65 and PH (r = 0.37). Significant but negative correlations were observed between GY on one hand, and ER, GLS, and TLB disease severity on the other (**Figure 1**). Under optimum management conditions, high positive and significant correlation was observed between PH and EH (r = 0.67) as well as between GLS and TLB disease severity (r = 0.66).

### GCA effects under artificial FAW condition and optimum conditions

The contribution of parental lines to the crosses were not consistent across traits and conditions. In experiment I, positive and significant GCA effects were observed for GY in CML312, CML567, CML488, DTPYC9-F46-1-2-1-2, CKDHL164288 and CKDHL166062 (**Table 6**). For GY in experiment II, only CLRCY039 showed positive and significant GCA effect. For foliar damage due to FAW, lines with negative GCA effects are preferred as lower score are associated with higher level of resistance to FAW infestation. For FD1, we observed three lines each with negative and significant GCA effects in both experiments. For FD2, four and three lines with significant and negative GCA effects were identified in experiment I and II, respectively, and for FD3, four parental lines were identified in each experiment. Only one parental line, CKDHL166068 showed consistent negative and significant GCA effects for foliar damage at the three times of assessment in experiment I. In experiment II, CLRCY039, CKDHL166087 and CKDHL166092 showed negative and significant GCA effects for foliar damage at all the three times of scoring. For ear damage, six parental lines in experiment I and five lines in experiment II showed negative and significant GCA effects. Parental lines that exhibited positive and significant GCA for GY and negative and significant GCA effects for resistance to at least one of FD1, FD2 and FD3, together with negative and significant GCA effects for ED, EXHL, ER and TLGTH are CML488 and CML567. In experiment II, CLRCY039 exhibited positive and significant GCA effects for GY trait as well as desirable (negative) and significant GCA effects for FD1, FD2, FD3, ED, EXHL, ER and TLGTH.

**Table 6:** General combining ability effects of the white maize inbred lines for grain yield and other traits under artificial infestation of FAW management.

Parents	GY	AD	PH	FD1	FD2	FD3	ED	EXHL	ER	TLGTH
Experiment I										
CML312	0.32**	0.54**	12.45**	0.05	-0.02	-0.15**	0.01	-0.12	4.12**	0.32
CKLTI0344	-0.28*	-1.93**	6.52**	-0.07	0.08	0.06	0.16	0.55**	2.29*	2.61**
CML488	0.47**	0.17	-2.17	-0.17**	-0.03	-0.05	-0.63**	-0.39*	-8.32**	-2.94
WMA2001	-0.01	-0.52**	-18.30**	-0.15**	-0.23**	0.06	-0.23*	-0.53**	0.72	-3.17**
DTPYC9-F46-1-2-1-2	0.24*	-2.11**	-3.05*	0.00	-0.14**	-0.13*	-0.36**	-0.19	-1.96	-1.74**
CKDHL164288	0.28*	0.51**	14.70**	0.18**	0.19**	-0.01	0.16	-0.06	-0.55	1.11*
CKDHL164260	-0.34**	-0.59**	5.14**	0.00	-0.01	-0.06	-0.09	0.06	-2.03	-0.55
CML567	1.11**	1.91**	3.21*	-0.01	-0.06	-0.32**	-0.27*	-0.33*	-4.08**	-0.64
KS23-6	-0.29**	0.64**	0.82	0.15**	0.15**	0.22**	0.69**	0.37*	7.46**	2.57**
CKLMARSI0183	-0.26*	-0.99**	-6.53**	0.11*	0.16**	-0.06	0.34**	0.30*	4.75**	1.60**
CKDHL166062	0.40**	1.76**	4.46**	-0.05	-0.03	0.23**	-0.32**	-0.03	-7.95**	-0.81
CKDHL166068	0.11	0.17	7.20**	-0.28**	-0.19**	-0.20**	0.34**	-0.12	3.02*	0.25
CKDHL121288	-0.66**	0.07	-12.98**	0.17**	0.13*	0.23**	0.61**	-0.04	9.58**	0.84
CKDHL166075	-0.28*	0.14	-9.50**	-0.02	-0.12*	0.00	-0.26**	0.34*	-3.06**	1.52**
CKDHL164271	-0.78**	0.23	-1.96	0.07	0.11*	0.19**	-0.15	0.18	-3.98**	-0.95
Experiment II										
CKDHL0089	0.12	0.55**	6.47**	0.08	0.16**	0.16**	-0.27**	0.11	-2.57**	0.15
CKDHL120348	0.23	0.50**	3.27	0.15*	0.20**	0.19**	0.34**	0.15	2.94**	1.26*
CKDHL120668	-0.26*	-0.93**	-7.70**	0.10*	0.14**	0.27**	-0.28**	-0.14	-0.20	-1.44**
CKDHL121320	-0.30*	0.90**	7.52**	0.00	-0.08	-0.13*	0.95**	0.29*	9.23**	2.53**
CKDHL166087	0.11	0.85**	0.59	-0.12*	-0.12*	-0.18**	0.44**	0.17	3.39**	1.81**
CKDHL166091	0.12	-0.12	-4.96*	-0.15*	-0.13*	-0.04	-0.34**	-0.12	-1.97*	0.38
CKDHL166092	-0.45**	-0.74**	-15.33**	-0.33**	-0.24**	-0.22**	-0.06	0.02	-2.04*	0.11
CKLMLN140377	-0.08	-1.50**	4.70*	0.07	0.07	-0.01	0.59**	0.01	4.52**	0.57
CKLMLN140538	0.07	-0.61**	-3.01	0.07	0.12*	0.21**	-0.38**	-0.26*	-1.29	-1.82**
CLRCY039	0.48**	0.18	14.25**	-0.12*	-0.18**	-0.21**	-1.49**	-0.45**	-14.40**	-3.81**
CML494	-0.05	0.93**	-5.80**	0.23**	0.05	-0.04	0.49**	0.23*	2.39*	0.26

*, and ** Significant at the 0.05 and 0.01 probability level, respectively. GY, grain yield; AD, days to anthesis; PH, plant height; FD1, FD2, FD3, mean foliar damage scores at 7, 14 and 21 days after artificial infestation, respectively; ED, ear damage; EXHL, number of exit holes; TLGTH, cumulative tunneling length; ER, number of rotten ears in %.

Under optimum conditions, in experiment I, parental lines that exhibited positive and significant GCA effects for GY are CML312, CKLTI0344, CKDHL164288, KS23-6 and CML567 (**Table 7**). Desirable negative and significant GCA effects for TLB and GLS disease resistance traits were observed for CKLTI0344, CML488 and CML567. Although WMA2001 had positive but non-significant GCA effects for GY, it exhibited negative (desirable) and significant GCA effects for PH, EH, ASI, TLB, and ER traits. In experiment II, CKDHL0089, CKDHL120348, CKLMLN140538 and CLRCY039 exhibited positive and significant GCA effects for GY. CKDHL120668, CKLMLN140538 and CLRCY039 exhibited desirable negative and significant GCA effects for TLB and GLS resistance traits. It is important that lines that show positive and significant GCA effect for GY under FAW infestation, together with at least one of significant negative GCA effect for FD1, FD2 and FD3, must also exhibit positive and significant GCA effect for GY in addition to negative and significant GCA effects for TLB and GLS under optimal conditions. Only two lines combine these effects as desired, and these are CML567 from experiment I and CLRCY039 from experiment II.

**Table 7:** General combining ability effects of the white maize inbred lines for grain yield and other traits evaluated under optimum management.

Parents	GY	AD	PH	EH	ASI	TLB	GLS	EPO	EPP	ER
Experiment I										
CML312	0.68**	0.60**	15.17**	4.11**	0.14**	0.12	-0.02	-0.02**	-0.02**	1.08**
CKLTI0344	1.04**	-1.12**	8.33**	3.77**	-0.32**	-0.83**	-0.81**	0.00	-0.01	-0.04
CML488	-0.56**	0.67**	-4.85**	-5.20**	-0.14*	-0.16*	-0.37**	-0.01**	0.01*	0.29
WMA2001	0.07	0.37**	-11.69**	-1.75**	-0.78**	-0.39**	-0.06	0.02**	0.01*	-0.91**
DTPYC9-F46-1-2-1-2	0.04	-3.01**	0.69	-1.93**	-0.76**	0.33**	0.37**	-0.01**	0.00	-1.27**
CKDHL164288	0.38**	0.51**	11.62**	2.91**	-0.16*	0.38**	0.66**	-0.01**	0.02**	-0.99**
CKDHL164260	-0.76**	-2.12**	9.14**	0.04	0.49**	0.25**	0.21**	-0.02**	0.01	-0.38
CML567	0.63**	2.68**	8.06**	7.69**	-0.51**	-0.54**	-0.60**	0.02**	0.04**	-2.15**
KS23-6	0.39**	0.01	-5.32**	-5.32**	0.72**	-0.08	0.17**	-0.01**	0.00	-1.99**
CKLMARSI0183	-0.24**	-1.40**	-3.51**	-9.58**	0.76**	0.53**	-0.26**	-0.03**	-0.02**	1.82**
CKDHL166062	0.00	1.71**	0.79	4.57**	-0.07	-0.11	-0.12*	0.02**	0.02**	-0.87*
CKDHL166068	-0.46**	0.86**	-2.42**	-0.62	0.13*	0.35**	0.40**	0.00**	-0.01	3.54**
CKDHL121288	-0.44**	1.20**	-11.69**	1.16**	-0.38**	0.23**	0.15*	0.03**	-0.02**	3.07**
CKDHL166075	-0.42**	-0.66**	-6.86**	-0.05	0.48**	-0.11	0.05	0.01**	-0.01*	0.20
CKDHL164271	-0.34**	-0.31**	-7.46**	0.18	0.39**	0.02	0.24**	0.01**	-0.03**	-1.41**
Experiment II										
CKDHL0089	0.60**	1.72**	-1.16	-3.18**	-0.76**	-0.18**	0.06	-0.01**	-0.01	0.97*
CKDHL120348	1.12**	0.84**	1.76*	5.98**	-0.44**	-0.33**	0.23**	0.02**	-0.01	0.19
CKDHL120668	-0.54**	-1.49**	-4.31**	-13.66**	0.00	-0.13**	-0.11**	-0.05**	-0.01	1.95**
CKDHL121320	-0.48**	0.42**	7.02**	9.36**	0.81**	0.04	-0.07*	0.02**	-0.04**	-2.00**
CKDHL166087	-0.34**	0.16	-0.18	5.76**	-0.43**	-0.05	-0.09*	0.02**	-0.04**	0.85*
CKDHL166091	-0.84**	-0.32**	-1.50*	0.45	0.00	0.08	0.10**	0.00	-0.01	-1.63**
CKDHL166092	-1.36**	-0.62**	-15.48**	-6.93**	0.70**	0.36**	-0.03	0.00	-0.06**	-0.83*
CKLMLN140377	0.07	-1.24**	7.75**	5.99**	0.11	0.26**	0.04	0.01**	0.05**	1.13**
CKLMLN140538	0.23**	-1.34**	-0.80	-8.42**	0.28**	-0.26**	-0.11**	-0.03**	0.01	0.90*
CLRCY039	1.88**	1.16**	6.67**	2.46**	0.28**	-0.14**	-0.14**	0.00	0.06**	-0.32
CML494	-0.32**	0.70**	0.25	2.19**	-0.56**	0.36**	0.13**	0.01**	0.04**	-1.21**

*, and ** Significant at the 0.05 and 0.01 probability level, respectively. GY, grain yield; AD, days to anthesis; ASI, anthesis to silking interval; PH, plant height; EH, ear height; TLB, turcicum leaf blight; GLS, gray leaf spot; EPO, ear position; EPP, ears per plant; ER, number of rotten ears in %.

### SCA effects under artificial FAW condition and optimum conditions

Under FAW infestation management in experiment I, 19 F1 hybrids showed significant SCA effects for GY, among which nine crosses had significant and positive SCA effects while the remaining 10 hybrids showed highly significant and negative SCA effects (**Supplementary Table S1**). In experiment II, 10 crosses had significant SCA effects for GY and among these, four crosses had positive SCA effects, while the SCA effects for the remaining crosses were negative. Under optimum conditions in experiment I, 38 crosses showed significant SCA effects for GY. Among these, 21 had positive SCA effects for GY while 17 showed negative SCA effects. In experiment II, 25 hybrids showed significant SCA effects for GY. Among these, 14 had positive SCA effect while 11 had negative SCA effect for GY (**Supplementary Table S1**). In both experiments, under FAW infestation and optimum conditions, seven crosses had significant and positive SCA effects for GY.

### Prediction of hybrid performance

**Figure 2:**
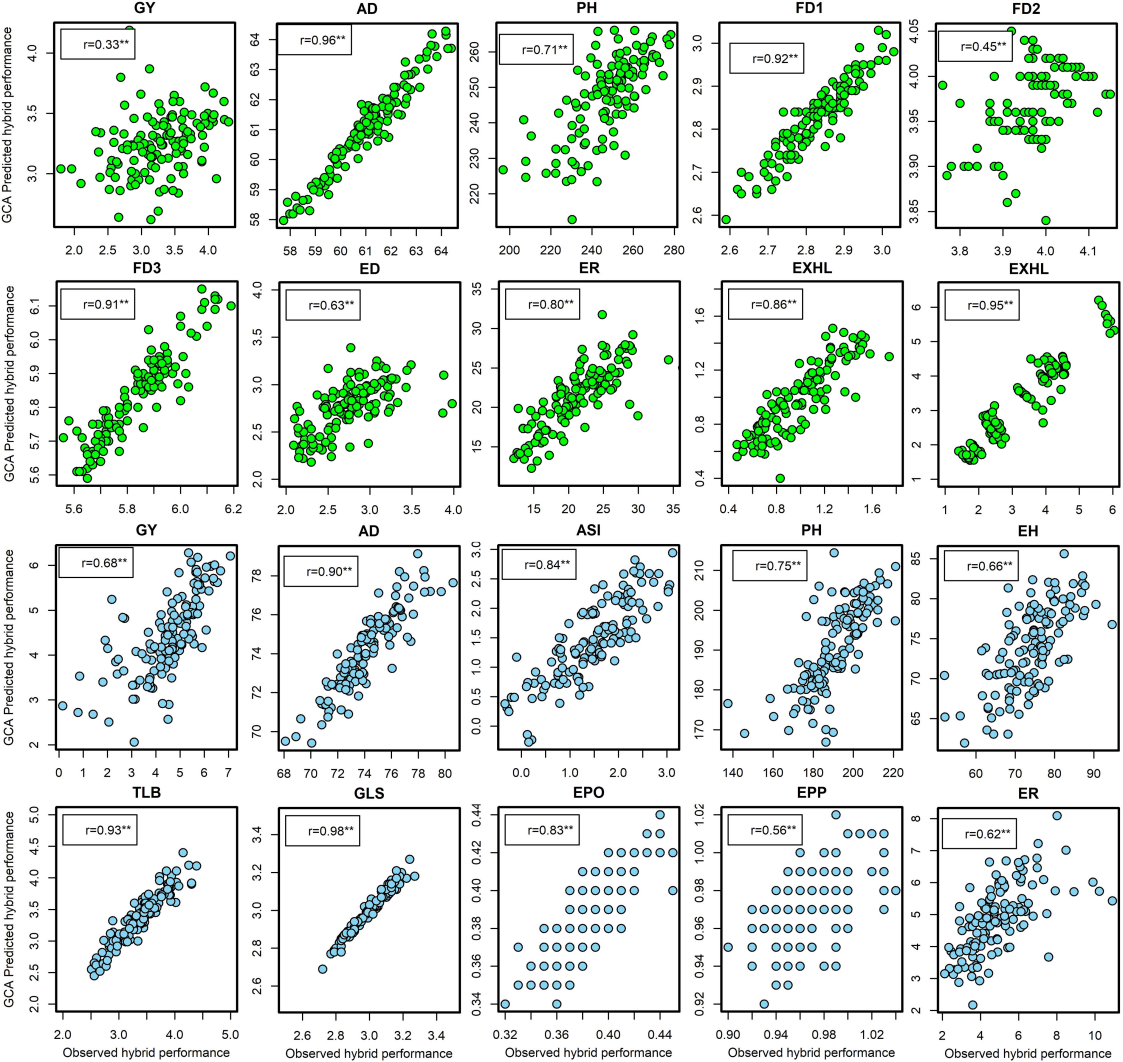
Leave-one-hybrid-out cross validated *r* values between general combining ability (GCA) based predicted hybrid performance and observed hybrid performance for grain yield and other agronomic traits evaluated under artificial infestation (in green dots) and under optimum management (blue dots) in three to five environments in experiment I. **Significant at the 0.01 probability level. GY, grain yield; AD, days to anthesis; PH, plant height; FD1, FD2, FD3, mean foliar damage scores at 7, 14 and 21 days after artificial infestation, respectively; ED, ear damage; EXHL, number of exit holes; TLGTH, cumulative tunneling length; ER, number of rotten ears in %. ASI, anthesis to silking interval; EH, ear height; TLB, turcicum leaf blight; GLS, gray leaf spot; EPO, ear position; EPP, ears per plant.

Under artificial FAW infestation in experiment I, the correlation coefficients between the observed hybrid performance and the predicted hybrid performance based on GCA ranged from 0.33 for GY to 0.86 for EXHL (**Figure 2**). Six traits viz. FD2, FD3, AD, ER, EXHL, and TLGTH were predicted with high accuracy as evidenced by their respective correlation coefficients of 0.92, 0.91, 0.96, 080, 0.86, and 0.95. Prediction efficiency was low for GY (0.33) but moderately high for FD2 (0.45) and ED (0.63). Similarly in experiment II (**Supplementary Figure S5**) the correlation between GCA-predicted and observed field performance of the hybrids was low for GY (r = 0.32, *P*<0.01) and FD2 (r = 0.30, *P*<0.01), moderate for EXHL (r = 0.56, *P*<0.01), PH (r = 0.58, *P*<0.01), FD3 (r = 0.57, *P*<0.01), TLGTH (r = 0.61, *P*<0.01) and AD (r = 0.66, *P*<0.01), and high for ED (r = 0.86, *P*<0.01) and ER (r = 0.72, *P*<0.01).

Under optimal conditions in experiment I, the correlation coefficients between GCA-based predicted and field values ranged from 0.56 (EPP) to 0.98 (GLS). Hybrid performance for traits such as AD, ASI, PH, GLS, and TLB was predicted with high accuracy (r = 84 to 98), whereas the predictive accuracy for traits such as GY, EPP, EPO, ER, EH, and PH was moderately high (r = 56 to 75; **Figure 2**). In experiment II (**Supplementary Figure S5**), the correlation values ranged from 0.42 (P<0.01) for GY to 0.97 (P<0.01) for EPO.

**Figure 3:**
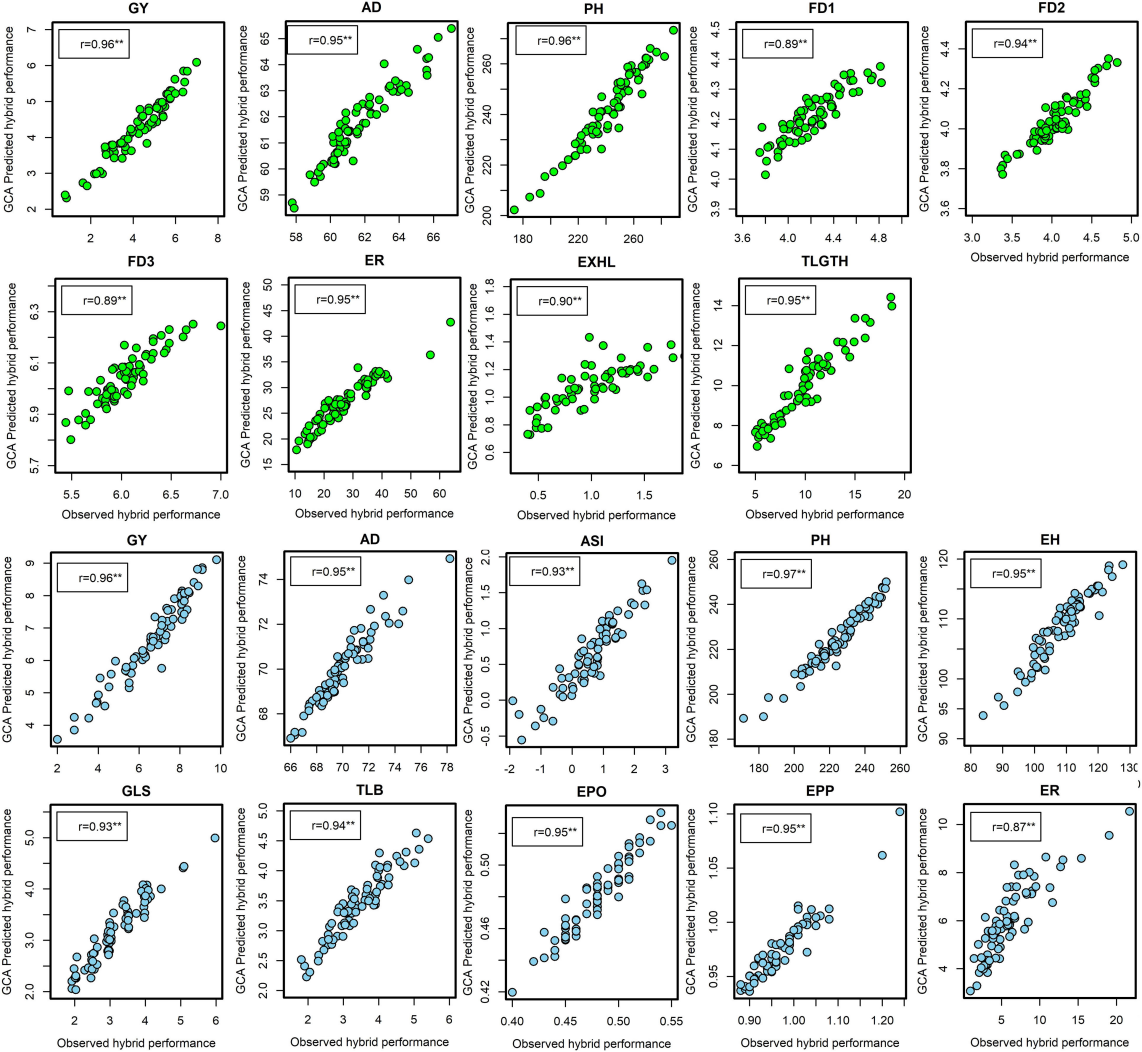
The correlation between marker-based predicted and observed F1 hybrid performance for grain yield and other agronomic traits evaluated under artificial infestation (in green dots) and under optimum management (blue dots) in three to five environments in experiment I. **Significant at the 0.01 probability level. ASI, anthesis to silking interval; ED, ear damage; EH, ear height; EPO, ear position; EPP, ears per plant; ER, ear rot; EXHL, number of exit holes; FD1, FD2, FD3, mean leaf damage scores 7,14 and 21 days after artificial infestation respectively; GLS, grey leaf spot.; GY, grain yield; PH, plant height; t/ha, tons per hectare; TLB, turcicum leaf blight; TLGTH, tunnel length.

Under artificial FAW infestation, the correlations between marker-based predicted and observed F_1_ hybrid performance was significant for all traits and were considerably higher than the correlation coefficients obtained between predicted hybrid performance based on GCA effects and the observed hybrid performance. Marker-based correlations with field performance for the hybrids ranged from 0.89 for FD3 to 0.96 for GY and PH (**Figure 3**). Under optimal conditions, correlation coefficients of prediction for all traits were significant and ranged from 0.87 for ER to 0.97 for PH. In effect, the prediction of hybrid performance using markers was more reliable for all agronomic traits, including complex traits such as GY and FAW resistance indicator traits.

## Discussion

Screening maize germplasm collections to identify multiple sources of FAW resistance trait is critical for successful FAW resistance breeding in SSA. Most of the hybrids evaluated in this study expressed moderate resistance to FAW foliar damage. Although none of the hybrids expressed a high resistant reaction to FAW leaf feeding damage, hybrids with partial resistance provide a valuable genetic resource that could be used to broaden the genetic base of the constricted breeding pool of FAW resistant maize and improve elite but FAW susceptible maize genotypes. Moderate resistance to FAW in maize was also reported by Ni et al. (2014) and Kasoma et al. (2021). According to Womack et al. (2018), many underlying genes whose effects are small confer partial resistance to FAW in maize. Polygenic resistance that confer partial but durable (Kliebenstein, 2017) protection has been reported for major lepidopteran pests that affect maize such as the African stem borers (Munyiri & Mugo, 2017), European corn borer (Foiada et al., 2015), and Southwestern corn borer (Khairallah et al., 1998). Insect resistance in maize has also been described as genetically broad-based and maize lines resistant to a particular insect-pest are also highly likely to be resistant to a different insect pest (Brooks et al., 2005). Improving the durability and stability of host plant resistance to FAW in maize by combining resistance traits from multiple germplasm sources, therefore, is critical as FAW could establish itself as a multi-generational pest of economic importance in SSA where climatic conditions are favorable and host plants are abundant (Prasanna et al., 2018).

In the current study, foliar damage scores increased with infestation duration from 4.9 at 7 days after artificial infestation to 5.9 at 21 days after infestation. This observation suggested that the post-infestation time threshold could be used to assess the level of foliar damage and guide the implementation of various FAW control actions such insecticide application which is a key component of the integrated pest management framework (van den Berg et al., 2021). FAW larvae mature within 2-3 weeks (Wan et al., 2021) and the high damage ratings observed 21 days after artificial infestation suggested that the most destructive FAW larva at late instar stages coincided with the most susceptible maize growth stage. Unlike mature plants, young maize seedlings at the 3-leaf stage (V3) to 5-leaf stage (V5) phenological stages of maize growth and development lack in their leaves the hard-to-digest cellulose and secondary metabolites that optimize the plant defense mechanisms against foliar herbivory (Bhoi et al., 2019). Results from this study revealed a significant but negative correlation between GY and foliar feeding damage scores at 7, 14, and 21 days after artificial FAW infestation. On average the top ten hybrids produced 25 and 2.3% more GY under optimum conditions than under FAW infested conditions in experiment I and II respectively. Low grain yield in maize under FAW infestation reported by previous studies (Abrahams et al., 2017; Assefa & Ayalew, 2019; Overton et al., 2021) corroborates our findings. In addition to direct loss of photosynthetic leaf area and alteration of the normal functioning of the remaining leaf tissue which stunts maize growth and development, FAW leaf feeding damage disrupts assimilate partitioning, resulting in poor grain filling and low yield (Buntin, 1986). FAW-inflicted kernel damage predisposes maize to fungal attacks, rots, and mycotoxin accumulation which reduce grain and seed quality (Williams et al., 2018). Maize genotypes resistant to the Mediterranean Corn Borer (MCB) have been used to reduce fumonisin contamination in maize kernels (Santiago et al. (2013). Similarly, deployment of effective host plant resistance to FAW is expected to reduce contamination of maize grain (Mahato et al., 2019; Pruter et al., 2021) with aflatoxins as well as the health burden associated with the treatment of disorders caused by chronic exposure to high levels of aflatoxin in humans and animals (Magnussen & Parsi, 2013).

Rating leaf and ear damage visually provided a quick and efficient method of discriminating FAW-susceptible from resistant lines and is therefore recommended for use in future insect-resistance screening. Ear damage trait showed moderately high heritability estimate (0.65 in experiment I and 0.51 in experiment II) when compared to that of FD1, FD2, and FD3 for which heritability ranged from 0.32 to 0.35 in experiment I and 0.38 to 0.49 in experiment II. These findings suggested that ED could be used as a selection index for FAW resistance.

Significant and negative correlation was observed between GY and FAW resistance indicator traits such as ED, ER, EXHL, and TLGTH. Stalk tunneling insects such as the FAW causes structural damage to the plant, disrupts water and nutrients flow, interferes with carbon source-sink translocation, and contributes to stem lodging which adversely affects the harvestable yield. Stem lodging attributed to tunneling by insect pest also constrain mechanical harvesting in large scale maize production. Significant and positive correlation observed between ED and ER (0.84) suggested that breeding for reduced ear damage due to FAW improves resistance to ear rot. Similarly, the positive correlation detected between GLS and TLB foliar diseases indicated the prospects of improving resistance to both diseases simultaneously.

Significant mean squares among hybrids for foliar damage scores at 7 days after artificial FAW infestation and other traits like GY, ED, ER, EXHL, TLGTH and AD suggested that there was adequate genetic variation for improving these traits in the set of germplasm studied. Non-significant mean squares observed for FD2 and FD3 traits in experiment I suggested that genotypes showed no differences at these stages of scoring. In both experiments, mean squares for hybrids were higher in all traits evaluated under optimal and FAW infestation conditions when compared to the mean squares obtained for hybrid by environment interaction (HxE). Although GXE is heritable, our results indicate a greater component for the genetic potential that is less influenced by environment. For GY under FAW infested conditions, the magnitude of the was 25%, 59% and 77% of the genetic variance observed among the parental lines. In the absence of epistasis, estimated for the lines reflect the additive genetic variation, which can be exploited in line or hybrid breeding in a recurrent manner. This observation is similar to findings by Beyene et al. (2011) and Karaya et al. (2009) that highlighted the preponderance of additive gene action for GY under stem borer infestation. Substantial magnitude of observed for GY under artificial FAW infestation in experiment II, in contrast to results obtained in experiment I, in addition to indicating that genetic effects are dependent on the germplasm used, implicate non-additive effects in the inheritance of resistance to FAW in maize. Consequently, breeding for resistance to FAW will benefit from heterotic grouping of parental lines. Kasoma et al. (2021), in their study, found that non-additive genetic effects were more important for GY under FAW infestation. Genotype x environment interaction was evident for most of the traits evaluated, including FAW resistance-associated traits, which indicates the importance of conducting multi-location trials to identify stable genotypes in contrasting environments (Abu et al., 2021). Farmers prefer hybrids with wide adaptation in target environments, where a combination of biotic and abiotic stresses occur frequently. Interactions of GCA and SCA with environment were significant for many traits under FAW infestation and optimal conditions, suggesting that the test environments were unique and influenced the combining abilities of the parental lines. These findings also underscore the possibility of developing hybrids that are adapted to specific environments. Detection of significant GxE interactions could also facilitate selection of genotypes that respond to target environments in a systematic and predictable manner (Prasanna et al., 2021).

Moderately high heritability estimates observed for GY under artificial FAW infestation conditions showed that yield-based selection could facilitate achievement of the desired genetic gains under conditions similar to those used in the present study. Under the two management conditions used, the ratio of GCA effects to the total genetic effects (2GCA+SCA) was closer to 1. These results indicate greater predictability of hybrid performance based on GCA effects. However, given the considerable magnitude of SCA effects, it is imperative to employ breeding strategies that also exploit this genetic effect to optimize performance under FAW- infested and optimal conditions. A total of 69 hybrids (across experiments I and II), representing 63% of all crosses, outyielded the best commercial check under FAW infestation, while 19 hybrids, amounting to 12%, outperformed the best check under optimal conditions. Hybrids that show resistance to FAW must also be capable of good productivity in FAW-free environments. In the current study, seven hybrids (4% of all hybrids evaluated) showed good performance under FAW-infested and optimal conditions. These hybrids have considerable potential for cultivation in areas prone to FAW infestation.

Lines that are parents of several top-performing hybrids under FAW and optimum management, such as, CML567 in experiment I, CKDHL120348, CKDHL166087 and CKDHL166091 in experiment II, have high frequency of favorable alleles for high yield in the presence or absence of FAW and demonstrate their potential for use in the development of high yielding hybrids with good performance under these conditions. Superior inbred lines for FAW resistance, GY, and resistance to TLB and GLS could be extracted from a population formed from CML567, CKDHL120348, CKDHL166087 and CKDHL166091. Combining ability analyses comparison across FAW infested and optimum conditions indicated that inbred lines CML312, CML567, CML488, DTPYC9-F46-1-2-1-2, CKDHL164288, CKDHL166062 and CLRCY039 showed significant and positive GCA effects for GY. On average, these lines contributed more to increase in yield in hybrid combinations under FAW infested conditions.

Parental lines CML567 and CKDHL166062 had the highest positive GCA effects for GY; in addition, they are also good general combiners for AD and PH (positive GCA effects) and for FD3, ED and ER (negative GCA effects). CML567 and CLRCY039 exhibited positive and significant GCA effect for GY under optimum and FAW infested conditions in addition to negative and significant GCA effects for TLB and GLS under optimal conditions. It is important to note that under optimum conditions, trials were left to natural GLS and TLB infestation and the uniformity of disease pressure could not be replicated. Further investigations are required to validate TLB and GLS disease resistance of CML567 and CLRCY039.

In hybrid maize development, multi-location evaluation of all possible cross combinations has practical limitations (Fasahat et al., 2016). Efficient methods are therefore required especially in resource constrained maize breeding programs to select for desirable traits among the parental lines and also predict hybrid performance in a time and cost-effective manner (Crossa et al., 2017). This is even more critical for traits like resistance to FAW and GY which require considerable resources and are complex in nature. In cross-pollinated crops like maize, parent lines of hybrids are usually selected based on their line *per se* performance and their GCA effects (Hallauer et al., 1988). The success of this approach depends on the correlation between hybrid performance and mean of the parental values or the sum of GCA effects of both parents. GCA-based predictions are promising for many traits in maize especially for traits predominantly controlled by additive effects (Nyaga et al., 2020). In this study, we obtained correlation greater than 0.60 between GCA-based prediction and field performance for traits like FD1, FD3, ED, ER, EXHL, and TLGTH under FAW artificial infestation management, and GY, AD, ASI, PH, EH, EPO, ER, TLB and GLS under optimum management conditions. One explanation for this high correlation is the high $\sigma_{2}^{\text{GCA}}$ observed for these traits, which is in agreement with earlier findings in maize (Schrag et al., 2009; Nyaga et al., 2020) and wheat (Longin et al., 2013). The $\sigma_{2}^{\text{GCA}}$ ratio varies depending on the allele frequencies between parental populations for the desired traits (Reif et al., 2007).

Our results showed higher accuracy in predicting hybrid performance with molecular data than GCA-based data. To accelerate genetic gains, several studies recommended the utilization of both phenotypic and genotypic data in predicting the performance of untested germplasm (Kadam et al., 2016; Wang et al., 2020). Predicting the performance of new hybrids based on marker data or estimates of GCA of inbred lines reduce labor and field expenditures associated with seed increases and multi-location evaluations of hybrids. Resource-constrained maize breeding programs in SSA could utilize marker-based predictions for identification and optimal selection of parents. This strategy is promising for accelerating development and deployment of superior yielding hybrids resistant to biotic and abiotic stresses that often occur together and frequently across maize growing agro-ecologies in SSA.

## Conclusions

Development of superior yielding, climate-resilient varieties resistant to FAW, GLS and TLB infestation is the key to sustainable maize production and food security in SSA. There was genetic variability for GY, FAW resistance traits, TLB and GLS and other agronomic traits among the 160 hybrids evaluated. Many inbred lines were identified with desirable GCA effect for GY, resistance to FAW and resistance to TLB and GLS. These inbred lines include CML312, CML567, CML488, DTPYC9-F46-1-2-1-2, CKDHL164288, CKDHL166062 and CLRCY039. The inbred lines have potential for use as parents of single cross and three-way cross hybrids and in the development of populations from which lines with resistance to multiple biotic stresses in SSA can be extracted. We identified several superior performing hybrids compared to best commercial checks for both GY and FAW resistance-associated traits. Both additive and non-additive gene action influence FAW resistance in the lines studied. Recurrent selection would be effective for the improvement of GY and resistance to FAW. The efficiency of hybrid maize breeding can be improved by prediction of the performances of untested single crosses based on the GCA performance of their parental inbred lines and marker data, with greater predictive ability achieved for models based on marker data. The exploitation and deployment of promising inbred lines and hybrids identified in this study have potential to mitigate the adverse effects of FAW, GLS and TLB infestation, boost maize productivity and contribute to food security in SSA.

## Data availability statement

The original contributions presented in the study are included in the article/**Supplementary Material**. Further inquiries can be directed to the corresponding authors.

## Author contributions

Conceptualization, funding acquisition, project and resources administration, P.B.M; methodology, investigations, formal analysis, and visualization, MG, FT, PP-R, BA, DM, YB, VA, JC, and IK; supervision, MG, BA and VA; Original draft preparation, IK; writing - review and editing; all authors. All authors contributed to the article and approved the submitted version.
