# Supplementary material for: Genetic analyses of tropical maize lines under artificial infestation of fall armyworm and foliar diseases under optimum conditions
Source: Front Plant Sci. 2023 Jan 20;14:1086757. doi: 10.3389/fpls.2023.1086757 (PMC9896009; doi:10.3389/fpls.2023.1086757)
Supplement: Supplementary file 1 [file DataSheet_1.docx]

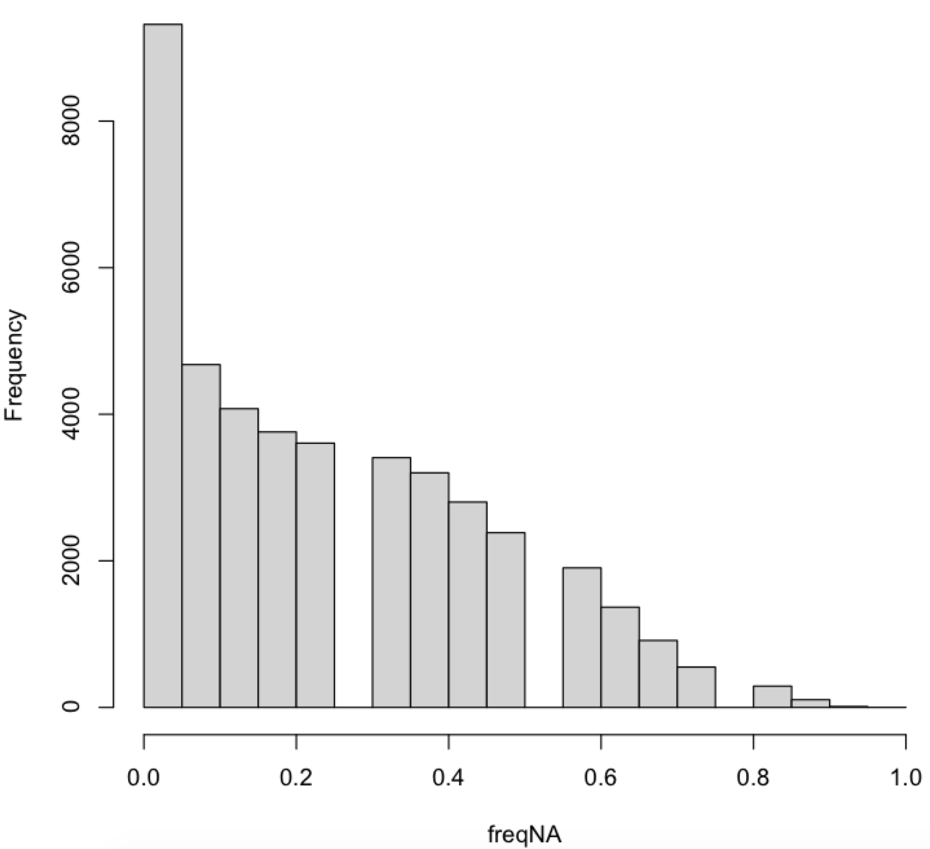


Supplementary Figure S1. Distribution of proportion of missing values from 42,376 SNPs in 13 parental lines.


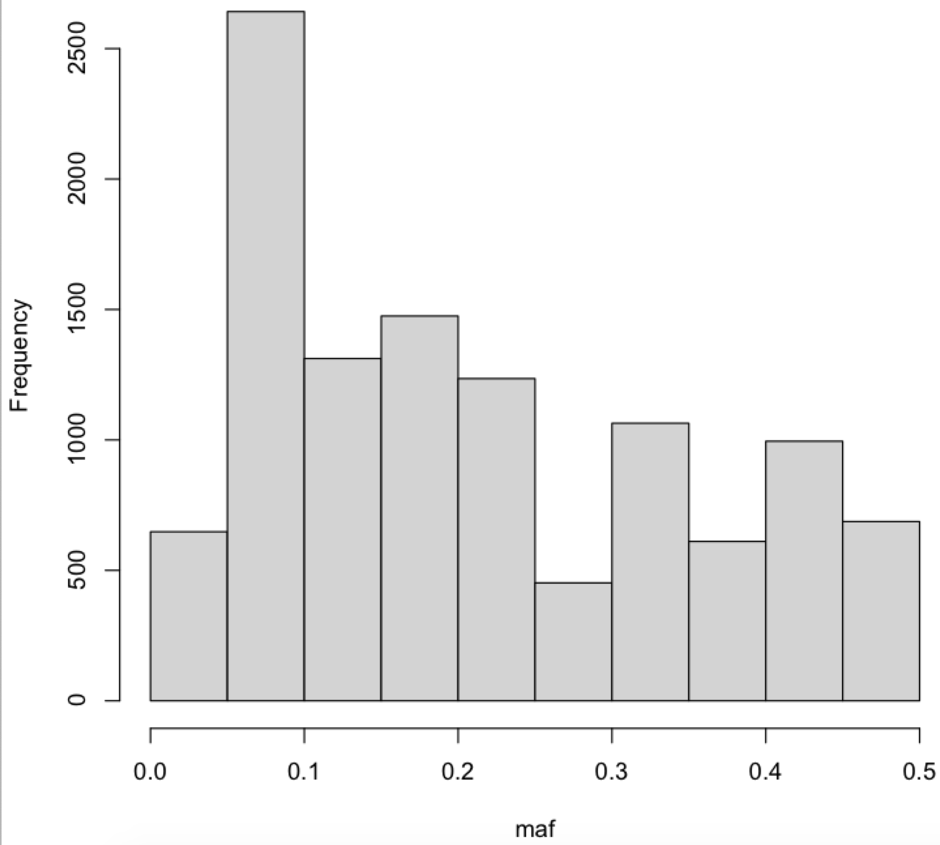


Supplementary Figure S2. Distribution of markers with different MAF.


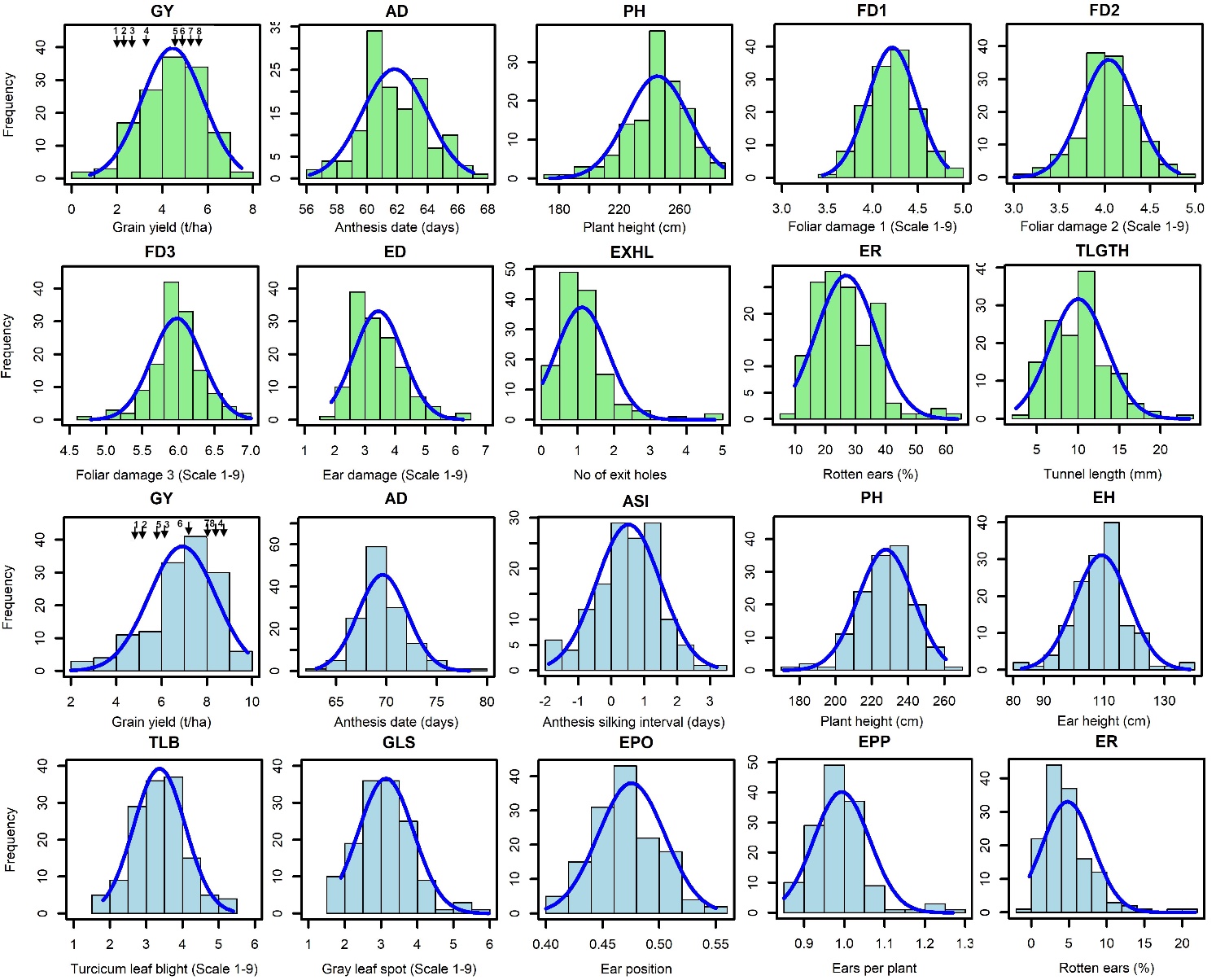


**Supplementary Figure S3.** Phenotypic distribution of traits evaluated under artificial infestation of fall army worm (green color) and optimum (blue color) management in experiment I. ASI, anthesis to silking interval; ED, ear damage; EH, ear height; EPO, ear position; EPP, ears per plant; ER, ear rot; EXHL, number of exit holes; FD1, FD2, FD3, mean leaf damage scores 7,14 and 21 days after artificial infestation respectively; GLS, grey leaf spot.; GY, grain yield; PH, plant height; t/ha, tons per hectare; TLB, turcicum leaf blight; TLGTH, tunnel length.


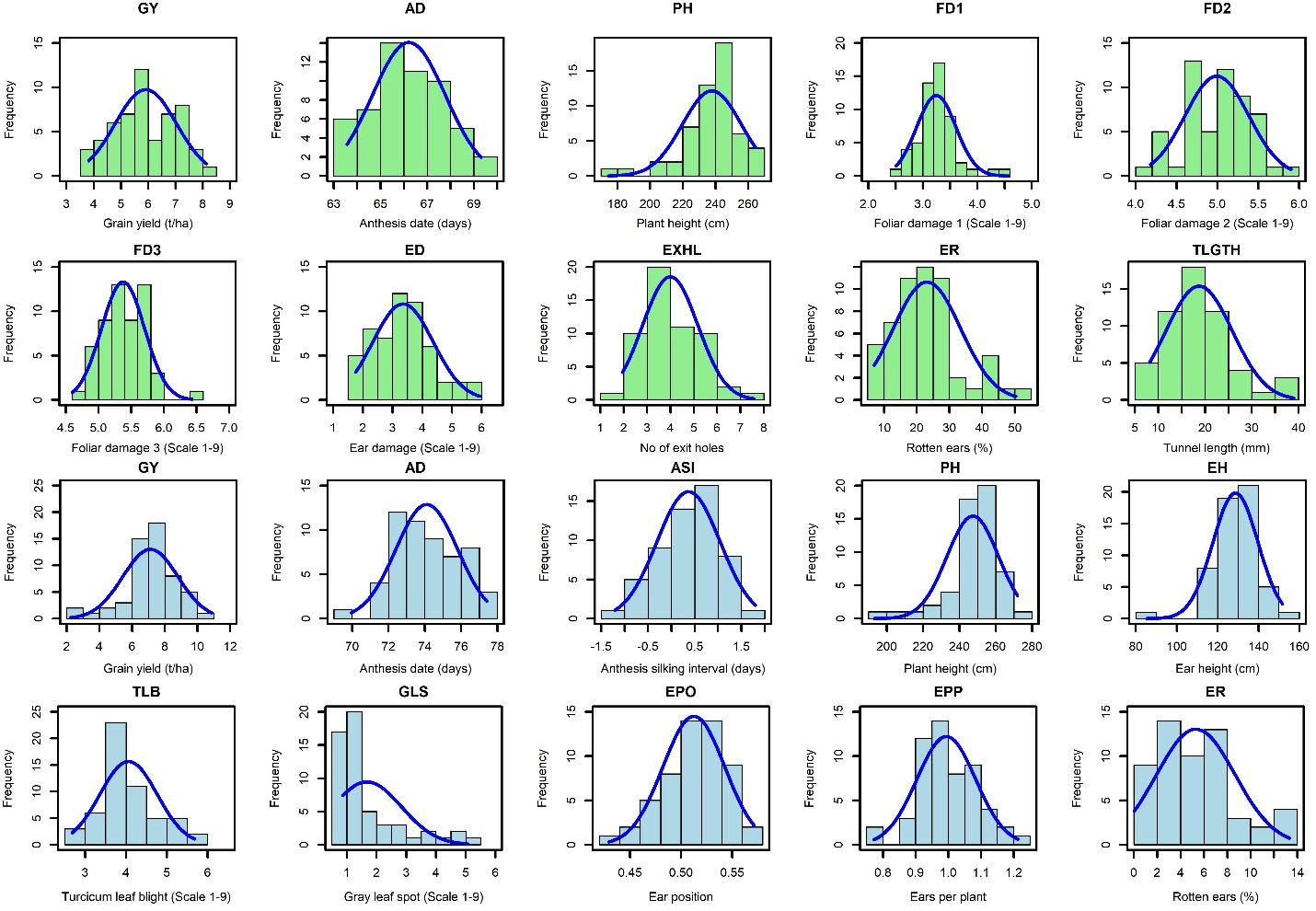


**Supplementary Figure S4.** Phenotypic distribution of traits evaluated under artificial infestation of fall army worm (green color) and optimum (blue color) management in experiment II. ASI, anthesis to silking interval; ED, ear damage; EH, ear height; EPO, ear position; EPP, ears per plant; ER, ear rot; EXHL, number of exit holes; FD1, FD2, FD3, mean leaf damage scores 7,14 and 21 days after artificial infestation respectively; GLS, grey leaf spot.; GY, grain yield; PH, plant height; t/ha, tons per hectare; TLB, turcicum leaf blight; TLGTH, tunnel length.


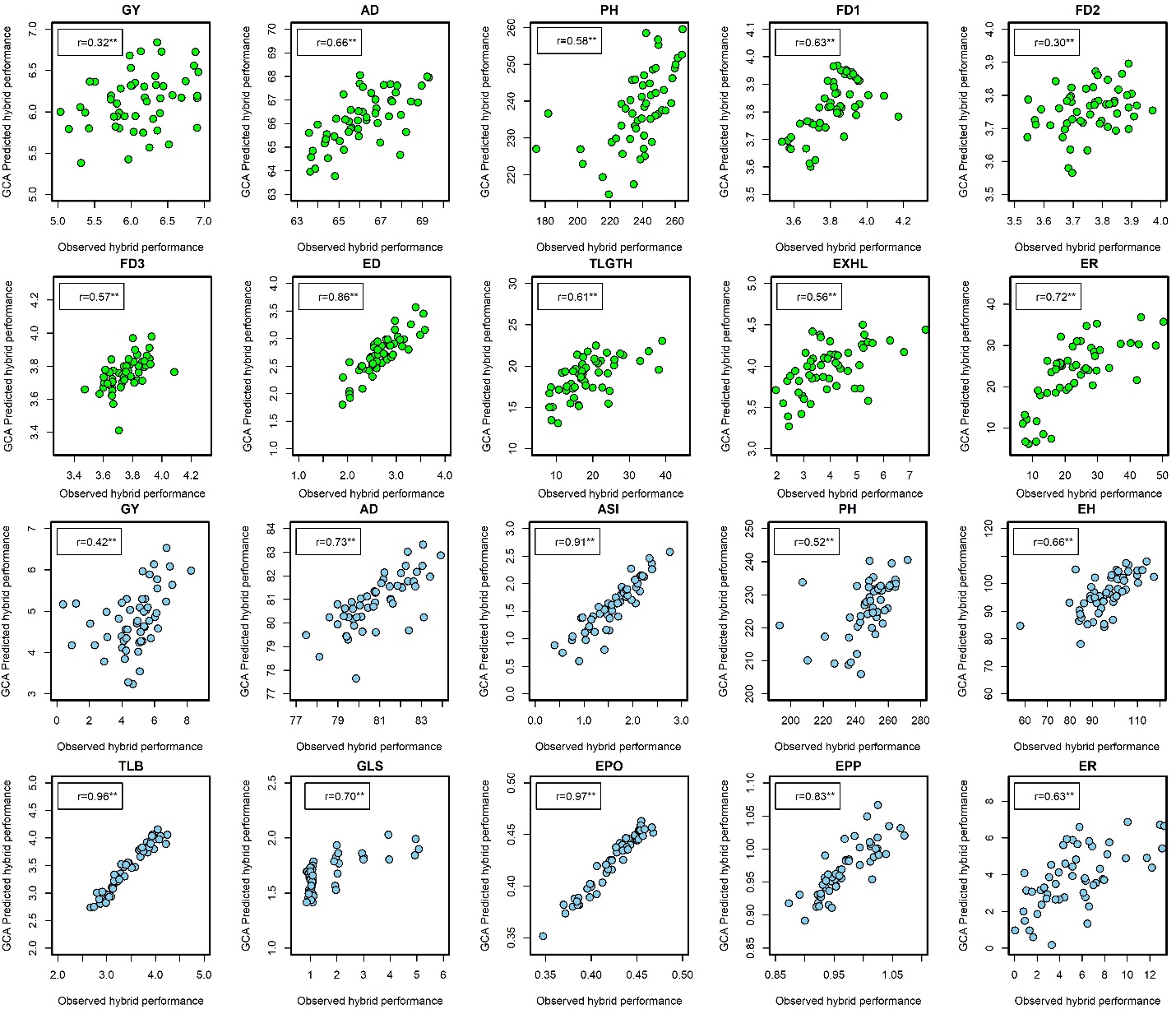


**Supplementary Figure S5.** Leave-one-hybrid-out cross validated *r* values between general combining ability (GCA) based predicted hybrid performance and observed hybrid performance for grain yield and other agronomic traits evaluated under artificial infestation (in green dots) and under optimum management (blue dots) in three to five environments in experiment II. **Significant at the 0.01 probability level. GY, grain yield; AD, days to anthesis; PH, plant height; FD1, FD2, FD3, mean foliar damage scores at 7, 14 and 21 days after artificial infestation, respectively; ED, ear damage; EXHL, number of exit holes; TLGTH, cumulative tunneling length; ER, number of rotten ears in %. ASI, anthesis to silking interval; EH, ear height; TLB, turcicum leaf blight; GLS, gray leaf spot; EPO, ear position; EPP, ears per plant.

Supplementary Table S1. SCA effects for selected traits evaluated under artificial FAW infestation and optimum conditions in experiment I and II.

| **______________FAW_______________ ____________Optimum___________** | | | | | | | | | |
| --- | --- | --- | --- | --- | --- | --- | --- | --- | --- |
| **Pedigree** | **GY** | **AD** | **FD2** | **FD3** | **ED** | **GY** | **AD** | **GLS** | **TLB** |
| **Experiment I** |  |  |  |  |  |  |  |  |  |
| CML312/CKDHL121288 | 1.85** | -0.60 | -0.25 | -0.21 | -0.78 | 0.61 | -0.82* | -0.54* | 0.77* |
| CKDHL164288/CKLMARSI0183 | 1.84** | -0.86 | -0.12 | -0.07 | -1.49*** | 1.17*** | -0.88** | -0.32 | -0.80* |
| CML312/CKDHL164271 | 1.71** | -0.77 | -0.01 | -0.22 | -0.26 | 1.15*** | -1.19*** | -0.14 | -1.03** |
| KS23-6-BBB/CKDHL166062 | 1.50** | -0.57 | 0.04 | 0.48 | -0.37 | -0.04 | 0.14 | 0.04 | -0.36 |
| CML312/CKDHL164288 | 1.49** | -0.39 | 0.14 | 0.21 | -0.32 | 0.64* | 1.12*** | 0.45 | 0.28 |
| CML488/CKDHL164260 | 1.39** | 1.42 | 0.47 | -0.08 | -0.33 | -1.07*** | 0.67* | -0.25 | 0.36 |
| CKLTI0344/DTPYC9-F46-1-2-1-2-BBB | 1.37** | -0.28 | -0.24 | -0.32 | 0.20 | 0.21 | 1.11*** | -0.48 | -0.05 |
| WMA2001/CKDHL164271 | 1.28* | -0.37 | 0.19 | 0.05 | -0.57 | 1.17*** | -0.08 | -0.10 | -0.18 |
| CKLTI0344/CKDHL164271 | 1.27* | -0.80 | 0.16 | -0.37 | -0.40 | 0.59 | -1.84*** | -0.35 | -0.41 |
| CKDHL164260/CML567 | 1.23 | -0.82 | -0.18 | 0.93*** | -0.22 | -0.12 | -0.21 | -0.02 | 0.41 |
| CKDHL164260/CKDHL166068 | 1.17 | -0.25 | -0.15 | 0.24 | -0.28 | 0.68* | -0.27 | 0.48 | -0.16 |
| CKDHL121288/CKDHL164271 | 1.16 | -0.80 | -0.25 | -0.25 | -0.96 | 1.32*** | -1.03*** | -0.31 | -0.47 |
| CML567/CKDHL164271 | 1.11 | -1.81 | 0.14 | 0.00 | -0.46 | 1.99*** | -1.52*** | -0.56* | 0.30 |
| CML567/KS23-6-BBB | 1.06 | -0.89 | -0.11 | 0.13 | -0.83 | -0.32 | 0.91** | 0.52 | 0.07 |
| DTPYC9-F46-1-2-1-2-BBB/CKDHL121288 | 1.05 | -1.95 | -0.32 | 0.38 | -0.58 | 1.08*** | -0.21 | 0.56 | 0.23 |
| WMA2001/CKDHL164288 | 0.98 | -0.82 | -0.09 | 0.00 | -0.38 | 0.57 | -1.02*** | -0.01 | -0.54 |
| DTPYC9-F46-1-2-1-2-BBB/CKLMARSI0183 | 0.93 | -2.90*** | 0.44 | -0.18 | -0.71 | -0.60 | -1.24*** | -0.03 | 0.25 |
| CML312/CKDHL166062 | 0.92 | -0.63 | 0.09 | 0.07 | -0.59 | 0.81* | -0.84* | -0.27 | -0.23 |
| CKDHL166068/CKDHL164271 | 0.89 | -0.64 | 0.20 | -0.48 | -1.27* | 0.52 | -0.22 | -0.37 | 0.20 |
| KS23-6-BBB/CKDHL121288 | 0.85 | 0.13 | 0.27 | 0.20 | 0.99 | 0.51 | 0.15 | -0.73* | -0.03 |
| CML312/CKLMARSI0183 | 0.82 | -2.22* | -0.60 | -0.22 | 0.17 | 0.94*** | 0.27 | -0.14 | -0.88* |
| CML488/CKDHL121288 | 0.79 | -0.23 | -0.11 | -0.33 | 0.02 | 0.54 | 0.61 | -0.19 | -0.29 |
| WMA2001/CKDHL166068 | 0.71 | -0.31 | 0.01 | 0.26 | -0.69 | -0.14 | 0.00 | 0.75** | 0.48 |
| DTPYC9-F46-1-2-1-2-BBB/CKDHL166068 | 0.69 | 1.11 | -0.08 | -0.07 | 0.17 | 0.49 | 1.75*** | 0.31 | -0.23 |
| CKDHL164288/CML567 | 0.68 | -0.26 | -0.34 | 0.33 | 1.86*** | 0.04 | -0.46 | 0.02 | 0.28 |
| CKDHL164288/CKDHL121288 | 0.66 | 0.09 | 0.30 | 0.21 | -0.17 | 1.68*** | -0.48 | 0.77** | 0.18 |
| WMA2001/CKDHL164260 | 0.63 | -1.72 | -0.01 | 0.11 | -0.49 | 0.89*** | -0.77* | 0.44 | -0.41 |
| CKLMARSI0183/CKDHL164271 | 0.62 | -0.32 | 0.04 | -0.19 | 0.39 | 0.76* | -1.09*** | 0.29 | -0.31 |
| CKDHL164260/CKDHL166062 | 0.61 | 1.00 | -0.36 | -0.36 | 0.08 | 0.54 | 0.51 | 0.00 | -0.36 |
| CML312/CML488 | 0.59 | -1.04 | 0.13 | -0.03 | -0.63 | 0.94*** | -1.42*** | 0.48 | 0.15 |
| DTPYC9-F46-1-2-1-2-BBB/CKDHL164288 | 0.56 | -0.40 | 0.17 | 0.17 | -0.62 | 0.12 | -0.77* | -0.28 | 0.07 |
| KS23-6-BBB/CKDHL164271 | 0.56 | -2.04* | -0.36 | -0.23 | -1.19* | 0.17 | 0.16 | 0.67* | -0.16 |
| CKLMARSI0183/CKDHL166062 | 0.52 | -1.60 | -0.14 | -0.11 | -0.32 | -0.26 | -0.46 | -0.54* | -0.64 |
| CML567/CKDHL166068 | 0.52 | -1.91* | 0.68* | -0.58* | -0.37 | 1.33 | -1.32*** | -0.21 | -0.03 |
| WMA2001/CML567 | 0.51 | 0.61 | 0.27 | -0.52 | -0.08 | -0.15 | 0.55 | -0.25 | -0.62 |
| CML312/CKDHL166075 | 0.49 | -1.68 | 0.08 | 0.15 | -0.50 | -0.31 | -0.08 | 0.06 | 0.10 |
| CML312/CKDHL164260 | 0.47 | -0.78 | -0.19 | 0.19 | -0.08 | 1.08*** | -0.51 | -0.10 | 0.07 |
| CKDHL164260/CKDHL121288 | 0.44 | -0.64 | 0.13 | -0.24 | -0.65 | 1.21*** | -1.23*** | -0.27 | -0.70 |
| DTPYC9-F46-1-2-1-2-BBB/CKDHL164271 | 0.43 | -0.95 | 0.56 | -0.15 | -0.04 | 0.40 | -1.82*** | 0.47 | 0.10 |
| CML488/CKDHL166068 | 0.41 | 1.33 | -0.25 | -0.18 | -0.18 | 0.33 | 1.07*** | 0.06 | -0.75* |
| CKLTI0344/CML567 | 0.40 | 0.52 | 0.20 | 0.54* | -0.22 | 0.28 | 0.42 | 0.50 | 0.48 |
| CKLTI0344/CML488 | 0.39 | -0.07 | -0.05 | 0.12 | -0.32 | -0.30 | -1.81*** | 0.77** | 0.43 |
| CKDHL164288/CKDHL166062 | 0.37 | -3.44** | 0.19 | -0.34 | -0.38 | 0.45 | 0.51 | -0.21 | -0.16 |
| WMA2001/DTPYC9-F46-1-2-1-2-BBB | 0.32 | 2.14* | -0.25 | 0.00 | 0.56 | -0.45 | 1.37*** | -0.23 | 0.18 |
| CKLTI0344/CKDHL164288 | 0.28 | -0.08 | -0.33 | -0.19 | -0.36 | 0.84* | -0.28 | -0.26 | -0.44 |
| KS23-6-BBB/CKDHL166075 | 0.27 | -0.95 | 0.05 | 0.31 | 0.37 | -0.30 | -0.36 | -0.14 | -0.03 |
| CML567/CKLMARSI0183 | 0.24 | 1.41 | -0.42 | -0.44 | -0.68 | 0.80 | -1.83*** | -0.06 | 0.12 |
| CKLTI0344/CKDHL166062 | 0.23 | -0.49 | -0.06 | 0.04 | -0.49 | 0.47 | -0.36 | 0.02 | -0.29 |
| CKLMARSI0183/CKDHL166068 | 0.22 | -0.85 | -0.03 | 0.31 | -0.11 | 0.87* | -1.11*** | -1.06*** | 0.89 |
| CKLTI0344/CKDHL166075 | 0.19 | -1.04 | -0.35 | 0.19 | 0.13 | 0.26 | -0.24 | -0.15 | -0.29 |
| CML312/CKDHL166068 | 0.19 | -0.04 | -0.13 | -0.21 | 0.05 | -0.92** | -0.49 | 0.21 | -0.03 |
| CML488/CKDHL164271 | 0.18 | -1.57 | -0.23 | 0.16 | -0.07 | 1.08** | -1.13*** | -0.79** | -0.08 |
| WMA2001/KS23-6-BBB | 0.15 | -0.12 | 0.21 | 0.13 | 0.36 | -0.06 | 0.35 | -0.02 | -0.75 |
| CML567/CKDHL166075 | 0.13 | -0.05 | 0.01 | -0.41 | -0.14 | 0.24 | -0.16 | 0.13 | -0.57 |
| CKDHL164288/KS23-6-BBB | 0.12 | -0.32 | -0.36 | -0.20 | -0.52 | -0.39 | -0.66* | -0.74** | 0.15 |
| CKDHL164288/CKDHL166068 | 0.09 | 0.15 | 0.08 | 0.01 | 0.16 | -0.21 | -0.89** | 0.02 | -0.62 |
| CML488/CML567 | 0.08 | 0.75 | 0.29 | -0.13 | -0.38 | 0.02 | 0.39 | 0.06 | -0.18 |
| CML488/CKDHL166062 | 0.04 | 1.58 | -0.16 | -0.05 | 0.40 | 0.37 | -0.50 | -0.42 | -0.29 |
| CKLMARSI0183/CKDHL121288 | -0.02 | -0.58 | 0.23 | -0.04 | -0.28 | -0.83* | -0.07 | 0.19 | 0.36 |
| CKDHL164260/KS23-6-BBB | -0.04 | 1.61 | -0.37 | -0.18 | -0.68 | -0.20 | 0.72* | 0.21 | 0.28 |
| CKDHL166062/CKDHL121288 | -0.05 | -0.59 | 0.33 | 0.10 | 0.26 | 0.32 | 0.91** | -0.19 | 0.20 |
| CKDHL166062/CKDHL164271 | -0.09 | 2.27* | 0.03 | -0.25 | -0.39 | 0.06 | 1.06*** | 0.15 | 0.00 |
| CKLTI0344/WMA2001 | -0.11 | 0.63 | 0.13 | 0.14 | 0.07 | 0.04 | 0.35 | -0.04 | 0.00 |
| DTPYC9-F46-1-2-1-2-BBB/CKDHL166075 | -0.12 | 1.31 | 0.09 | 0.08 | 0.32 | -0.01 | 0.03 | 0.16 | -0.44 |
| CKDHL166068/CKDHL121288 | -0.14 | -0.32 | -0.23 | 0.16 | 1.90*** | -0.70* | -0.55 | 0.96*** | 1.53*** |
| DTPYC9-F46-1-2-1-2-BBB/CKDHL164260 | -0.14 | -0.63 | 0.20 | 0.10 | 0.29 | 0.29 | -1.27*** | 0.01 | 0.20 |
| CKDHL164260/CKDHL166075 | -0.15 | -0.89 | -0.26 | -0.09 | -0.10 | 0.44 | 0.39 | -0.17 | 0.30 |
| CML567/CKDHL166062 | -0.16 | -0.67 | -0.01 | 0.24 | -0.29 | -0.17 | 0.08 | 0.31 | 0.10 |
| CKLTI0344/CKDHL164260 | -0.16 | -0.31 | -0.17 | -0.19 | 0.13 | 0.01 | 0.34 | -0.31 | 0.02 |
| CKLTI0344/CKDHL121288 | -0.17 | 0.70 | 0.01 | -0.04 | 0.15 | -0.06 | 0.40 | 0.00 | 0.38 |
| WMA2001/CKDHL166075 | -0.19 | -0.12 | -0.12 | -0.13 | -0.23 | -0.82* | -1.39*** | -0.40 | 0.61 |
| CML488/CKDHL164288 | -0.22 | 2.99*** | -0.12 | 0.06 | 0.00 | 0.45 | 0.42 | -0.21 | 0.56 |
| CKDHL121288/CKDHL166075 | -0.22 | 1.29 | 0.26 | -0.23 | 0.43 | -0.45 | 0.69* | -0.12 | -0.34 |
| CKLTI0344/CKLMARSI0183 | -0.44 | -0.58 | 0.22 | 0.23 | 0.45 | 0.01 | -0.88** | 0.65* | 0.07 |
| CML488/CKDHL166075 | -0.46 | -1.31 | 0.38 | 0.38 | 0.57 | -0.93** | 1.45*** | 0.40 | 0.38 |
| DTPYC9-F46-1-2-1-2-BBB/CKDHL166062 | -0.46 | 0.52 | 0.18 | 0.01 | -0.52 | 0.05 | 0.41 | -0.67* | -0.44 |
| CML567/CKDHL121288 | -0.47 | -1.14 | -0.10 | 0.19 | 0.05 | 0.67* | 0.35 | -0.46 | -0.90* |
| KS23-6-BBB/CKDHL166068 | -0.47 | 0.69 | 0.03 | 0.23 | 0.49 | 0.47 | -0.52 | 0.02 | 0.18 |
| CKLTI0344/CKDHL166068 | -0.53 | 0.10 | -0.23 | 0.12 | -0.02 | 0.09 | 0.36 | 0.00 | -0.41 |
| CKDHL164260/CKLMARSI0183 | -0.58 | 1.08 | 0.18 | 0.08 | 0.52 | 0.25 | 0.24 | 0.13 | 0.00 |
| CML312/WMA2001 | -0.58 | -0.01 | 0.14 | -0.16 | -0.36 | 0.03 | -0.37 | 0.17 | 0.71* |
| WMA2001/CKDHL121288 | -0.59 | 0.13 | -0.20 | 0.21 | -0.14 | -0.40 | 0.66* | 0.00 | 0.28 |
| CML488/CKLMARSI0183 | -0.59 | -0.35 | 0.02 | 0.06 | -0.34 | -0.31 | -0.80** | 0.21 | 0.07 |
| CKDHL166068/CKDHL164290 | -0.62 | -1.23 | -0.34 | 0.11 | -0.19 | -0.05 | -1.07*** | -0.06 | 0.41 |
| CKDHL166075/CKDHL164271 | -0.64 | 1.13 | -0.16 | 0.81 | 0.46 | 0.44 | -0.85** | -0.21 | -0.13 |
| CML488/KS23-6-BBB | -0.68 | 0.52 | -0.17 | -0.36 | 0.84 | 0.28 | 1.42*** | -0.21 | 0.02 |
| DTPYC9-F46-1-2-1-2-BBB/KS23-6-BBB | -0.69 | 0.47 | -0.18 | 0.12 | -0.43 | -0.24 | -0.89 | 0.54 | 0.20 |
| CKDHL164288/CKDHL166075 | -0.73 | 1.01 | -0.25 | -0.14 | -0.04 | 0.07 | 0.76* | 0.37 | 0.51 |
| CKLTI0344/KS23-6-BBB | -0.80 | -0.54 | 0.30 | -0.37 | -0.08 | -0.67* | -0.41 | -0.27 | 0.36 |
| CML488/WMA2001 | -0.84 | -2.81*** | -0.04 | 0.09 | 0.14 | -1.24** | 0.19 | 0.02 | -0.67 |
| CML312/KS23-6-BBB | -0.90 | 1.65 | 0.25 | -0.34 | 0.27 | 0.69* | -1.01*** | -0.06 | -0.26 |
| KS23-6-BBB/CKLMARSI0183 | -0.94 | 0.34 | 0.40 | 0.07 | 0.79 | 0.10 | -0.01 | 0.17 | 0.33 |
| CML488/DTPYC9-F46-1-2-1-2-BBB | -1.09 | -1.22 | -0.13 | 0.29 | 0.27 | -0.16 | -0.56 | 0.08 | 0.28 |
| WMA2001/CKLMARSI0183 | -1.12 | 1.01 | -0.18 | -0.17 | 1.03 | 0.37 | 0.26 | -0.10 | 0.64 |
| WMA2001/CKDHL166062 | -1.16 | 1.77 | -0.05 | -0.01 | 0.77 | 0.18 | -0.10 | -0.23 | 0.28 |
| CML312/DTPYC9-F46-1-2-1-2-BBB | -1.32* | 1.41 | -0.05 | 0.26 | 0.82 | -0.22 | 0.89** | -0.26 | -0.34 |
| CKDHL166062/CKDHL166068 | -1.48* | 6.42*** | -0.05 | 0.67 | 0.58 | -3.28** | 7.60*** | 0.60 | -0.11 |
| DTPYC9-F46-1-2-1-2-BBB/CML567 | -1.53* | 1.37 | -0.40 | -0.70 | 0.27 | -0.98** | 1.18*** | -0.19 | 0.00 |
| CKDHL164260/CKDHL164271 | -1.79* | 0.86 | 0.29 | -0.60* | 0.36 | -1.41** | 1.03*** | -0.37 | -0.16 |
| CML312/CKLTI0344 | -1.93*** | 2.23 | 0.42 | 0.09 | 0.75 | -1.80*** | 2.83*** | -0.08 | 0.15 |
| CKDHL166062/CKDHL120566 | -2.13*** | 1.18 | 0.15 | 0.02 | -0.07 | -2.08** | -0.81* | 1.06*** | 0.66 |
| CKDHL164288/CKDHL164271 | -3.04*** | 2.25 | 0.30 | -0.15 | 0.84 | -2.85** | 2.28*** | 0.18 | 0.38 |
| CKDHL164288/CKDHL164260 | -3.08*** | 0.08 | 0.42 | 0.10 | 1.43 | -2.59*** | 0.34 | 0.22 | 0.15 |
| CKDHL166068/CKDHL166075 | -3.21*** | 2.43 | -0.12 | 0.14 | 1.97 | -3.79** | 1.79*** | 0.04 | -0.13 |
| CML312/CML567 | -3.81*** | 2.88 | -0.02 | 0.42 | 1.48 | -3.64*** | 1.60*** | 0.21 | 0.53 |
| **Experiment II** |  |  |  |  |  |  |  |  |  |
| CKDHL0089/CKDHL120348 | -0.06 | 0.90 | 0.13 | 0.14 | 0.27 | 0.32 | 0.24 | 0.05 | 0.15 |
| CKDHL0089/CKDHL120668 | -0.88 | 0.16 | 0.05 | -0.20 | 0.55 | 0.38 | -0.13 | -0.11 | -0.22 |
| CKDHL0089/CKDHL121320 | -0.81 | 0.32 | -0.08 | 0.16 | -0.30 | -0.62* | -0.04 | -0.15 | -0.06 |
| CKDHL0089/CKDHL166087 | -1.48* | 0.38 | 0.03 | 0.07 | 0.37 | -1.34*** | 1.22* | 0.03 | 0.04 |
| CKDHL0089/CKDHL166091 | 0.71 | 0.02 | 0.08 | 0.16 | -0.44 | -1.69*** | 0.29 | 0.01 | -0.09 |
| CKDHL0089/CKDHL166092 | -0.17 | 0.31 | -0.02 | -0.11 | -0.60 | -1.09*** | 0.29 | 0.14 | -0.20 |
| CKDHL0089/CKLMLN140377 | 1.39** | -0.43 | -0.09 | 0.11 | -0.62 | 1.47*** | -0.68 | -0.10 | 0.22 |
| CKDHL0089/CKLMLN140538 | 1.12 | -0.49 | 0.01 | -0.02 | 0.19 | 0.68* | 0.42 | 0.05 | 0.24 |
| CKDHL0089/CLRCY039 | -0.21 | 0.88 | -0.22 | -0.27 | 0.32 | 1.22*** | -0.48 | 0.09 | -0.04 |
| CKDHL0089/CML494 | 0.38 | -2.04** | 0.10 | -0.04 | 0.27 | 0.67 | -1.12* | -0.02 | -0.04 |
| CKDHL120348/CKDHL120668 | 0.40 | -0.45 | -0.10 | -0.02 | -0.22 | 0.13 | 0.05 | 0.05 | -0.07 |
| CKDHL120348/CKDHL121320 | -1.56 | 1.55* | 0.46* | 0.43 | -0.75* | 0.47 | 0.64 | 0.01 | 0.09 |
| CKDHL120348/CKDHL166087 | 1.93** | -1.73 | 0.26 | 0.02 | -0.19 | -0.13 | 0.29 | 0.20 | 0.19 |
| CKDHL120348/CKDHL166091 | 0.21 | -0.59 | -0.24 | -0.28 | 0.18 | -0.21 | 0.17 | 0.35 | 0.06 |
| CKDHL120348/CKDHL166092 | 0.37 | -1.64* | -0.15 | 0.10 | 0.09 | 0.55 | -1.13* | -0.02 | 0.11 |
| CKDHL120348/CKLMLN140377 | -0.90 | 1.95** | 0.03 | -0.10 | 0.34 | 0.41 | -0.71 | -0.26 | -0.13 |
| CKDHL120348/CKLMLN140538 | 0.03 | -1.43 | -0.15 | -0.12 | 0.15 | -0.67* | -1.21* | -0.28 | -0.11 |
| CKDHL120348/CLRCY039 | 0.30 | 1.44 | -0.03 | -0.26 | -0.02 | -1.00** | 1.49** | -0.08 | -0.06 |
| CKDHL120348/CML494 | -0.73 | 0.02 | -0.21 | 0.11 | 0.15 | 0.14 | 0.16 | -0.02 | -0.22 |
| CKDHL120668/CKDHL121320 | 0.09 | -0.53 | -0.36 | -0.27 | -1.02** | 0.14 | 0.17 | 0.01 | -0.11 |
| CKDHL120668/CKDHL166087 | 1.55** | -0.81 | 0.10 | 0.06 | -0.97** | 1.37*** | 0.63 | 0.03 | -0.02 |
| CKDHL120668/CKDHL166091 | -0.06 | -1.00 | 0.07 | -0.01 | 0.23 | 0.68* | 0.50 | -0.15 | 0.19 |
| CKDHL120668/CKDHL166092 | 0.05 | -0.21 | -0.62** | -0.64** | -0.09 | 2.09*** | -2.40*** | -0.02 | -0.26 |
| CKDHL120668/CKLMLN140377 | -1.06 | 1.05 | -0.02 | -0.44* | 0.41 | 0.13 | -0.27 | 0.07 | -0.17 |
| CKDHL120668/CKLMLN140538 | -0.85 | 3.49*** | 0.58** | 0.71** | 1.19** | -3.71*** | 2.43*** | 0.22 | 0.35* |
| CKDHL120668/CLRCY039 | 0.64 | -0.97 | 0.33 | 0.84*** | 0.51 | -1.11*** | -0.57 | 0.09 | 0.41* |
| CKDHL120668/CML494 | 0.12 | -0.72 | -0.04 | -0.04 | -0.58 | -0.09 | -0.41 | -0.19 | -0.09 |
| CKDHL121320/CKDHL166087 | -0.81 | 1.03 | -0.05 | -0.09 | 1.52*** | -2.19*** | 1.52** | 0.00 | -0.19 |
| CKDHL121320/CKDHL166091 | -0.42 | 1.17 | -0.14 | -0.17 | 0.58 | 0.19 | -0.71 | -0.02 | 0.02 |
| CKDHL121320/CKDHL166092 | -0.42 | 1.62* | 0.25 | 0.36 | 1.54*** | -2.65*** | 2.69*** | 0.27 | 0.24 |
| CKDHL121320/CKLMLN140377 | 0.37 | -1.95** | 0.06 | 0.11 | 0.40 | 0.96** | -1.08* | 0.03 | 0.17 |
| CKDHL121320/CKLMLN140538 | 1.88** | -0.18 | 0.36 | 0.16 | -0.97** | 1.55*** | -1.08* | 0.01 | 0.02 |
| CKDHL121320/CLRCY039 | 1.16 | -0.64 | -0.54** | -0.66** | -0.60 | 0.95** | -0.78 | 0.05 | 0.07 |
| CKDHL121320/CML494 | 0.50 | -2.39** | 0.04 | -0.03 | -0.40 | 1.20*** | -1.32** | -0.23 | -0.26 |
| CKDHL166087/CKDHL166091 | -1.35* | 0.89 | -0.21 | 0.08 | 0.52 | 0.41 | -1.65** | -0.17 | -0.22 |
| CKDHL166087/CKDHL166092 | -1.19* | 0.68 | 0.31 | 0.36 | 0.47 | -0.90** | 1.05* | -0.21 | 0.00 |
| CKDHL166087/CKLMLN140377 | 1.01 | -1.06 | -0.29 | 0.07 | -0.77 | 1.30*** | -0.93 | -0.11 | -0.07 |
| CKDHL166087/CKLMLN140538 | -0.24 | -0.12 | 0.11 | -0.12 | -1.07** | 0.73** | -0.43 | 0.03 | 0.11 |
| CKDHL166087/CLRCY039 | 0.78 | -1.42* | 0.18 | 0.08 | -0.36 | -0.30 | -0.63 | 0.07 | -0.17 |
| CKDHL166087/CML494 | -0.20 | 2.17** | -0.45 | -0.53* | 0.48 | 1.05*** | -1.06* | 0.13 | 0.33* |
| CKDHL166091/CKDHL166092 | 0.78 | -0.85 | 0.23 | 0.09 | -0.76* | 0.17 | -0.97 | -0.06 | 0.04 |
| CKDHL166091/CKLMLN140377 | -0.08 | -0.76 | 0.04 | -0.10 | -0.27 | -0.21 | 1.15* | 0.03 | -0.04 |
| CKDHL166091/CKLMLN140538 | 0.30 | 0.52 | 0.15 | 0.21 | 0.30 | 0.04 | 0.45 | -0.15 | -0.19 |
| CKDHL166091/CLRCY039 | -1.24* | 1.05 | 0.16 | -0.15 | 0.39 | 0.08 | 1.05* | -0.11 | 0.04 |
| CKDHL166091/CML494 | 1.16 | -0.43 | -0.15 | 0.17 | -0.74* | 0.54 | -0.28 | 0.27 | 0.20 |
| CKLMLN140377/CKDHL166092 | 0.27 | -0.47 | -0.27 | -0.27 | -0.63 | 0.71* | -1.05* | 0.00 | 0.19 |
| CKLMLN140538/CKDHL166092 | -0.12 | -0.53 | -0.34 | -0.16 | 0.20 | 0.34 | 0.35 | 0.14 | 0.04 |
| CLRCY039/CKDHL166092 | 0.57 | -0.66 | 0.40 | 0.39 | -0.09 | -0.08 | 0.05 | 0.01 | -0.07 |
| CML494/CKDHL166092 | -0.15 | 1.76* | 0.20 | -0.12 | -0.14 | 0.86** | 1.12 | -0.26 | -0.07 |
| CKLMLN140377/CKLMLN140538 | 0.70 | -0.10 | -0.28 | -0.36 | -0.27 | 0.20 | -0.43 | -0.10 | -0.20 |
| CKLMLN140377/CLRCY039 | -0.31 | -0.23 | 0.07 | 0.30 | -0.43 | -0.38 | 0.77 | -0.06 | -0.15 |
| CKLMLN140377/CML494 | -1.39* | 2.02** | 0.75*** | 0.68** | 1.84*** | -4.60 | 3.24*** | 0.50 | 0.19 |
| CKLMLN140538/CLRCY039 | -2.40*** | -0.12 | -0.28 | -0.19 | 0.72* | 0.62 | -0.53 | 0.09 | -0.13 |
| CKLMLN140538/CML494 | -0.42 | -1.04 | -0.16 | -0.10 | -0.43 | 0.23 | 0.04 | -0.02 | -0.13 |
| CLRCY039/CML494 | 0.72 | 0.67 | -0.08 | -0.09 | -0.44 | 0.01 | -0.36 | -0.15 | 0.09 |

*, and ** Significant at the 0.05 and 0.01 probability level, respectively. GY, grain yield; AD, days to anthesis; FD2, FD3, mean foliar damage scores at 14 and 21 days after artificial infestation, respectively; ED, ear damage; GLS, gray leaf spot; TLB, Turcicum leaf blight.
